# Daily defined dose-costs have a stronger influence on antibacterial drug prescriptions in Germany than bacterial resistance: economic factors are more important than scientific evidence

**DOI:** 10.1007/s00210-024-03435-7

**Published:** 2024-09-20

**Authors:** Lilly Josephine Bindel, Roland Seifert

**Affiliations:** Institute of Pharmacology, Hannover Medical School, 30625 Hannover, Germany

**Keywords:** Antimicrobial consumption, Antibiotic, antibacterial drug, Antimicrobial prescription, Antibacterial resistance, Antibiotic prescription, Germany, Arzneiverordnungsreport, Surveillance, Antibiotic stewardship, Correlation, Rational prescribing behavior, Irrational prescribing behavior

## Abstract

Previous research from our group revealed a strong association between daily defined dose (DDD)-costs and -prescriptions of antibacterial drugs in Germany (https://pubmed.ncbi.nlm.nih.gov/38842562/; https://pubmed.ncbi.nlm.nih.gov/39042156/). These data indicate that low costs are major driver of high prescription numbers. This study examines the relationship between bacterial resistance and DDD-prescriptions of antibacterial drugs using data from the Arzneiverordnungsreport (AVR) from 2008 to 2022 and the Antibiotic Resistance Surveillance (ARS) statistics provided by the Robert Koch Institute (RKI). We hypothesized that semi-rational or irrational prescribing behavior of antibacterial drugs is evident in Germany, i.e., prescriptions are driven to a greater extents by low DDD-costs than bacterial resistance. A bivariate correlation analysis was performed to test these models. Our data point to irrational prescribing behavior for amoxicillin, cefuroxime axetil, doxycycline, nitrofurantoin, ciprofloxacin, and clarithromycin. For amoxicillin clavulanic acid and sulfamethoxazole-trimethoprim, data point to semi-rational prescribing. For no antibacterial drug, a model pointing to rational drug prescribing was found. In conclusion, our study shows that DDD-costs exert a more significant influence on DDD-prescriptions than bacterial resistance, indicating that economic factors, rather than scientific evidence, primarily drive antibacterial drug prescriptions in outpatient settings in Germany. It will be important to conduct similar studies on the prescription of antibacterial drugs in other countries. It will also be important to study the relation between DDD-costs and -prescriptions for other drug classes and assess the scientific basis for these relations.

**Supplementary Information:**

The online version contains supplementary material available at 10.1007/s00210-024-03435-7.

## Introduction

The effectiveness and availability of antibacterial drugs have come under significant threat in recent years, primarily due to the rising incidence of bacterial resistance (WHO 2023) and the emergence of supply bottlenecks (BfArM 2023). Several factors contribute to this situation, including patterns of drug consumption, the development of bacterial resistance and economic considerations (Bindel and Seifert 2024a). To effectively address these challenges, it is crucial to understand the mechanisms and parameters that influence antibacterial drug use.

**Fig. 1:**
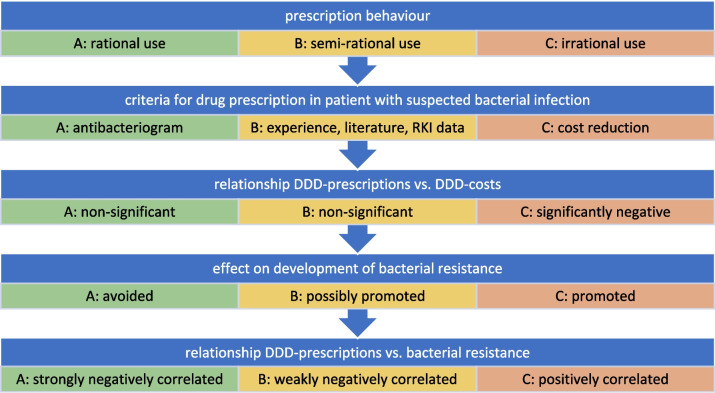
Hypothesis and characterization for prescription behavior of antibacterial drugs in relation to the correlation between DDD-costs and bacterial resistance with DDD-prescriptions. Green color is used to highlight characteristics of an optimal, rational use. Yellow color is used for a semi-rational use. Red color highlights a problematic, irrational use

Among various factors, the prescribing behavior of physicians plays an important role in the long-term effectiveness of antibacterial drugs. We propose three categories of prescribing behavior: rational, semi-rational, and irrational (see Fig. 1). Rational prescribing is guided by an individual antibacteriogram, ensuring that treatment decisions are based on specific bacterial sensitivities. In contrast, semi-rational prescribing relies on personal experience, literature, and data from the Robert Koch Institute (RKI). Irrational prescribing, however, prioritizes cost reduction for the healthcare system, disregarding factors such as bacterial resistance, which are central to rational and semi-rational decision-making.

These different models for prescribing behaviors have distinct implications for the development of bacterial resistance. Rational prescribing aims to minimize the development of resistance, while semi-rational prescribing may inadvertently promote resistance due to limited pathogen-specific knowledge. Irrational prescribing is particularly problematic, as it is likely to accelerate the spread of resistance (Sweileh 2021) by ignoring crucial factors in the decision-making process. Additionally, the relationship between DDD-costs and -prescriptions varies: it is weak or negligible in rational and semi-rational prescribing but is strongly correlated with irrational prescribing behavior.

Our categorization of prescribing behavior provides a framework for understanding real-world patterns by analyzing correlations between DDD-prescriptions and other relevant parameters. Previous research has shown a significant negative correlation between DDD-costs and -prescriptions (Bindel and Seifert 2024b), which suggests a prevalence of irrational or semi-rational prescribing (Retnosari et al. 2017). Regarding the correlation between bacterial resistance development and DDD-prescriptions, a negative correlation would indicate rational or semi-rational behavior, as an increase in resistance would lead to reduced use of the affected drug. Conversely, a positive correlation would suggest irrational behavior, where resistance increases due to frequent use of an antibacterial drug without a corresponding reduction in its prescription.

The aim of this study is to assess the influence of bacterial resistance on DDD-prescriptions to characterize the prescribing behavior for the ten most frequently prescribed antibacterial drugs in Germany in recent years. In addition, a unique comparison of the parameters DDD-costs and bacterial resistance has been performed to allocate the influence on DDD-prescriptions.

## Materials and methods

### Data collection of DDD-prescriptions

The analysis of DDD-prescriptions of antibacterial drugs is based on the Arzneiverordnungsreport (AVR, Drug prescription report) spanning from 2008 to 2022 (Schwabe and Paffrath 2010a, b, 2011a, b, 2012, 2013, 2014, 2015, 2016, 2017; Schwabe et al. 2018, 2019; Schwabe and Ludwig 2020; Ludwig et al. 2021, 2023, 2024). Since we focused on outpatient prescriptions, only the general chapter “Antibiotika und Chemotherapeutika” (antibiotics and chemotherapeutics) was considered. Therefore, specialized subchapters like urology, dermatology, and ophthalmology were excluded.

### Data collection of bacterial resistance

Bacterial resistance data was sourced from the Antibiotic Resistance Surveillance (ARS, https://ars.rki.de/) statistics provided by the Robert Koch Institute (RKI), covering the years since 2008 (RKI 2024).

Various options had to be selected. We chose the outpatient care area and included all material groups, regions, and specializations. Given were the values for sensitive, intermediate, and resistant pathogens in percent. We only took into account the percentage of resistant pathogens.

### Selection of drugs

The study focused exclusively on antibacterial drugs. We identified the top 15 most prescribed drugs in 2022: amoxicillin, cefuroxime axetil, doxycycline, amoxicillin-clavulanic acid, clindamycin, azithromycin, phenoxymethylpenicillin, sulfamethoxazole-trimethoprim, nitrofurantoin, ciprofloxacin, clarithromycin, cefaclor, cefpodoxime, pivmecillinam, and roxithromycin.

A lack of data availability for resistance statistics led to the exclusion of phenoxymethylpenicillin and the drugs ranked 12–15. The final list comprised ten drugs: amoxicillin, cefuroxime axetil, doxycycline, amoxicillin-clavulanic acid, clindamycin, azithromycin, sulfamethoxazole-trimethoprim, nitrofurantoin, ciprofloxacin, and clarithromycin.

### Analysis of correlations and presentation of data

Correlation analysis between bacterial resistance and DDD-prescriptions was conducted using SPSS, with a bivariate correlation analysis employing the Pearson correlation coefficient. The coefficient of determination (*R*^2^) was calculated manually. Data processing and visualization were performed using SPSS and Excel.

Correlation analysis included bacterial resistance vs. DDD-prescriptions, as well as a comparison of the parameters bacterial resistance and DDD-costs towards DDD-prescriptions. We did not include correlations between pathogens.

During the analysis, we emphasized three aspects of the correlations: significance, direction (positive vs. negative), and strength. The Pearson coefficient indicates whether there is a linear relationship between the two variables. The correlation coefficient ranges from − 1 to + 1. A positive coefficient indicates that both variables influence each other in the same direction, while a negative coefficient indicates an inverse relationship. A value of 0 signifies no linear relationship, while a value of 1 indicates a very strong linear relationship with same proportions of growth (Mukaka 2012). We define a correlation above (+ / −) 0.8 as strong, indicating a substantial influence between both factors. Values below 0.8 suggest a weaker relationship.

Beside the correlation coefficient, the significance of the correlation should be considered. The significance level indicates the extent to which the values can be generalized and considered reliable. Only significant values validate the correlation coefficient, allowing to draw conclusions. If there is non-significance, the values are only limited informative. A value with a significance level of 0.01 as well as 0.05 is determined to be considered significant.

The coefficient of determination (*R*^2^), calculated by squaring the Pearson correlation coefficient, indicates the proportion of the variance in the dependent variable that is predictable from the independent variable. Beside the significance, it can be used as an indicator whether the given correlation is valid.

Figure 2 illustrates the methodological procedure. Figures 3, 4, and 5 and Tables 1, 2, 3, and 4 show the most important results of our study. Supplemental Tables S1-S10 and supplemental Figures S1-S5 present some more detailed analysis.

**Fig. 2:**
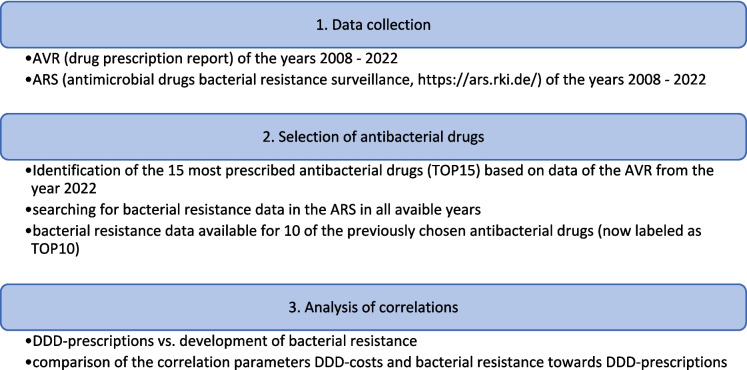
Metholodical approach of the selection and analysis of correlations regarding antibacterial drugs

## Results

### Correlation of bacterial resistance and DDD-prescriptions for amoxicillin

**Fig. 3:**
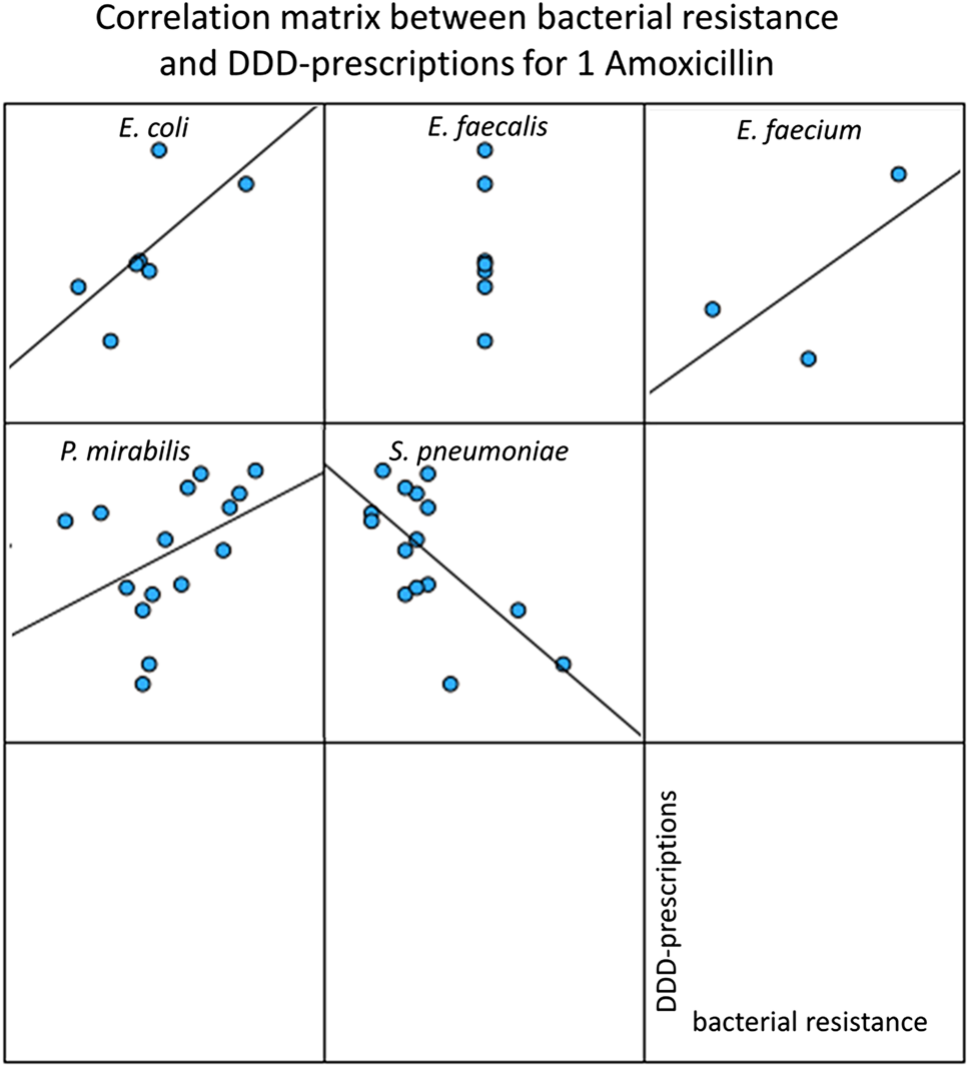
Correlation panels between bacterial resistance and DDD-prescriptions for amoxicillin. For the years 2008 to 2022, the development of bacterial resistance is plotted on the *x*-axis and DDD-prescriptions are plotted on the *y*-axis

Amoxicillin belongs to the substance group of aminopenicillins and is the most prescribed antibacterial drug in 2022 (Ludwig et al. 2024). The bacterial resistance data was provided for five pathogens: *E. coli*, *E. faecalis*, *E. faecium*, *P. mirabilis*, and *S. pneumoniae*. Data can be found in Table S1 as well as a graphical visualization in Fig. 3.

Significant correlations between bacterial resistance and DDD-prescriptions were observed for *E. coli* (0.679) and *S. pneumoniae* (− 0.663) (Table 1). However, neither correlation was strong, as the correlation coefficients did not exceed (+ / −) 0.8. Non-significant correlations were noted for *E. faecalis*, *E. faecium*, and *P. mirabilis*.

**Table 1:** Summary of correlation parameters between bacterial resistance of available pathogens and DDD-prescriptions for analyzed antibacterial drugs. Values in bold indicate a significant correlation at the 0.01 level. Values in italic indicate a significant correlation at the 0.05 level. Values in bold italic indicate no significant correlation

Pathogen	Correlation parameters	1 Amoxicillin	2 Cefuroxime axetil	3 Doxycycline	4 Amoxicillin clavulanic acid	5 Clindamycin	6 Azithromycin	7 Sulfamethoxazole-Trimethoprim	8 Nitrofurantoin	9 Ciprofloxacin	10 Clarithromycin
*E. coli*	Pearson correlation	**0.679**	*0.570*	-	***0.457***	-	-	*0.627*	*0.557*	**0.922**	-
	Sig. (2-tailed)	0.005	0.026		0.086			0.012	0.031	0	
	Coefficient of determination	0.461	0.325		0.002			0.393	0.31	0.85	
*A. baumanii*	Pearson correlation	-	-	-	-	-	-	-	-	**0.690**	-
	Sig. (2-tailed)									0.004	
	Coefficient of determination									0.476	
*C. freundii*	Pearson correlation	-	-	-	-	-	-	− ***0.232***	-	***0.001***	-
	Sig. (2-tailed)							0.468		0.997	
	Coefficient of determination							0.054		0	
*E. cloacae*	Pearson correlation	-	-	-	-	-	-	− **0.800**	-	**0.731**	-
	Sig. (2-tailed)							0.002		0.002	
	Coefficient of determination							0.64		0.534	
*E. faecalis*	Pearson correlation	− ***0.546***	-	-	***0.365***	-	-	-	− ***0.666***	***0.019***	-
	Sig. (2-tailed)	0.161			0.3				0.05	0.945	
	Coefficient of determination	0.298			0.133				0.444	0	
*E. faecium*	Pearson correlation	***0.694***	-	-	***0.937***	-	-	-	-	− ***0.561***	-
	Sig. (2-tailed)	0.511			0.063					0.621	
	Coefficient of determination	0.482			0.878					0.315	
*C. oxytoca*	Pearson correlation	-	− ***0.045***	-	− ***0.456***	-	-	− ***0.341***	-	**0.811**	-
	Sig. (2-tailed)		0.872		0.08			0.277		0	
	Coefficient of determination		0.002		0.208			0.116		0.658	
*K. pneumoniae*	Pearson correlation	-	*0.555*	-	***0.464***	-	-	− *0.688*	-	− ***0.151***	-
	Sig. (2-tailed)		0.032		0.081			0.013		0.59	
	Coefficient of determination		0.308		0.215			0.473		0.023	
*M. morganii*	Pearson correlation	-	-	-	-	-	-	− ***0.367***	-	− ***0.484***	-
	Sig. (2-tailed)							0.241		0.068	
	Coefficient of determination							0.135		0.234	
*P. mirabilis*	Pearson correlation	***0.417***	***0.088***	-	*0.528*	-	-	-	− ***0.418***	− ***0.454***	-
	Sig. (2-tailed)	0.122	0.002		0.043				0.156	0.089	
	Coefficient of determination	0.174	0.008		0.279				0.175	0.206	
*P. aeruginosa*	Pearson correlation	-	-	-	-	-	-	− ***0.425***	-	***0.390***	-
	Sig. (2-tailed)							0.167		0.151	
	Coefficient of determination							0.181		0.152	
*S. marcescens*	Pearson correlation	-	-	-	-	-	-	-	-	***0.153***	-
	Sig. (2-tailed)									0.586	
	Coefficient of determination									0.023	
*S. aureus*	Pearson correlation	-	− ***0.001***	**0.737**	− **0.946**	− **0.745**	− ***0.085***	***0.256***	− ***0.197***	**0.858**	**0.752**
	Sig. (2-tailed)		0.997	0.002	0	0.001	0.763	0.527	0.481	0	0.001
	Coefficient of determination		0	0.543	0.895	0.555	0.007	0.066	0.039	0.736	0.566
*S. epidermidis*	Pearson correlation	-	***0.455***	***0.140***	− **0.761**	− *0.607*	***0.341***	− ***0.342***	− ***0.187***	**0.817**	**0.733**
	Sig. (2-tailed)		0.088	0.619	0.001	0.016	0.214	0.408	0.505	0	0.002
	Coefficient of determination		0.207	0.02	0.579	0.368	0.116	0.117	0.035	0.667	0.537
*S. pneumoniae*	Pearson correlation	− **0.663**	− ***0.23***	*0.603*	**0.762**	***0.298***	***0.357***	-	-	-	***0.358***
	Sig. (2-tailed)	0.007	0.41	0.017	0.001	0.281	0.346				0.190
	Coefficient of determination	0.44	0.053	0.364	0.581	0.089	0.127				0.128

### Correlation of bacterial resistance and DDD-prescriptions for cefuroxime axetil

**Fig. 4:**
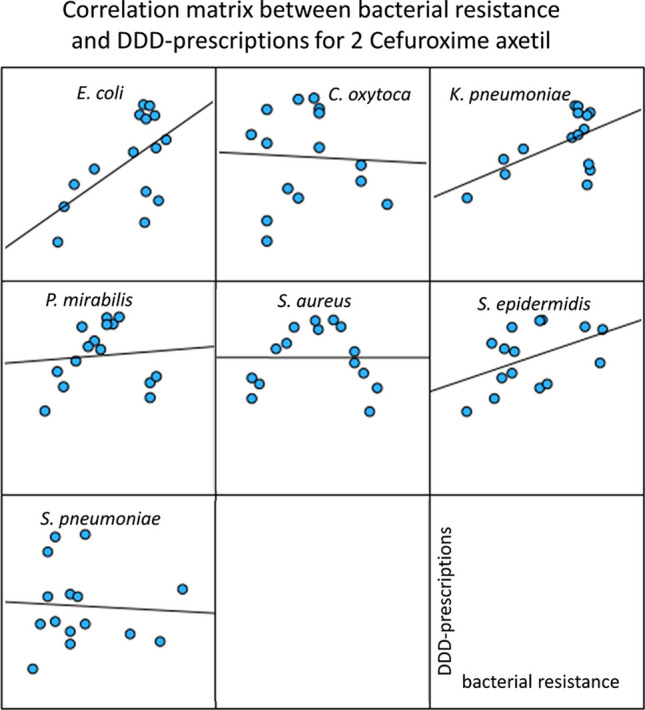
Correlation panels for the correlations between bacterial resistance and DDD-prescriptions for cefuroxime axetil. For the years 2008 to 2022, the development of bacterial resistance is plotted on the *x*-axis and DDD-prescriptions are plotted on the *y*-axis

Cefuroxime axetil belongs to the substance group of cephalosporins and is the second most prescribed antibacterial drug in 2022 (Ludwig et al. 2024). Resistance data included seven pathogens: *E. coli*, *C. oxytoca*, *K. pneumoniae*, *P. mirabilis*, *S. aureus*, *S. epidermidis*, and *S. pneumoniae*. Data can be found in Table S2 as well as a graphical visualization in Fig. 4.

Significant positive correlations between bacterial resistance and DDD-prescriptions were found for *E. coli* (0.570) and *K. pneumoniae* (0.555), though neither was strong. Non-significant correlations were observed for *C. oxytoca*, *P. mirabilis*, *S. aureus*, *S. epidermidis*, and *S. pneumoniae*.

### Correlation of bacterial resistance and DDD-prescriptions for doxycycline

**Fig. 5:**
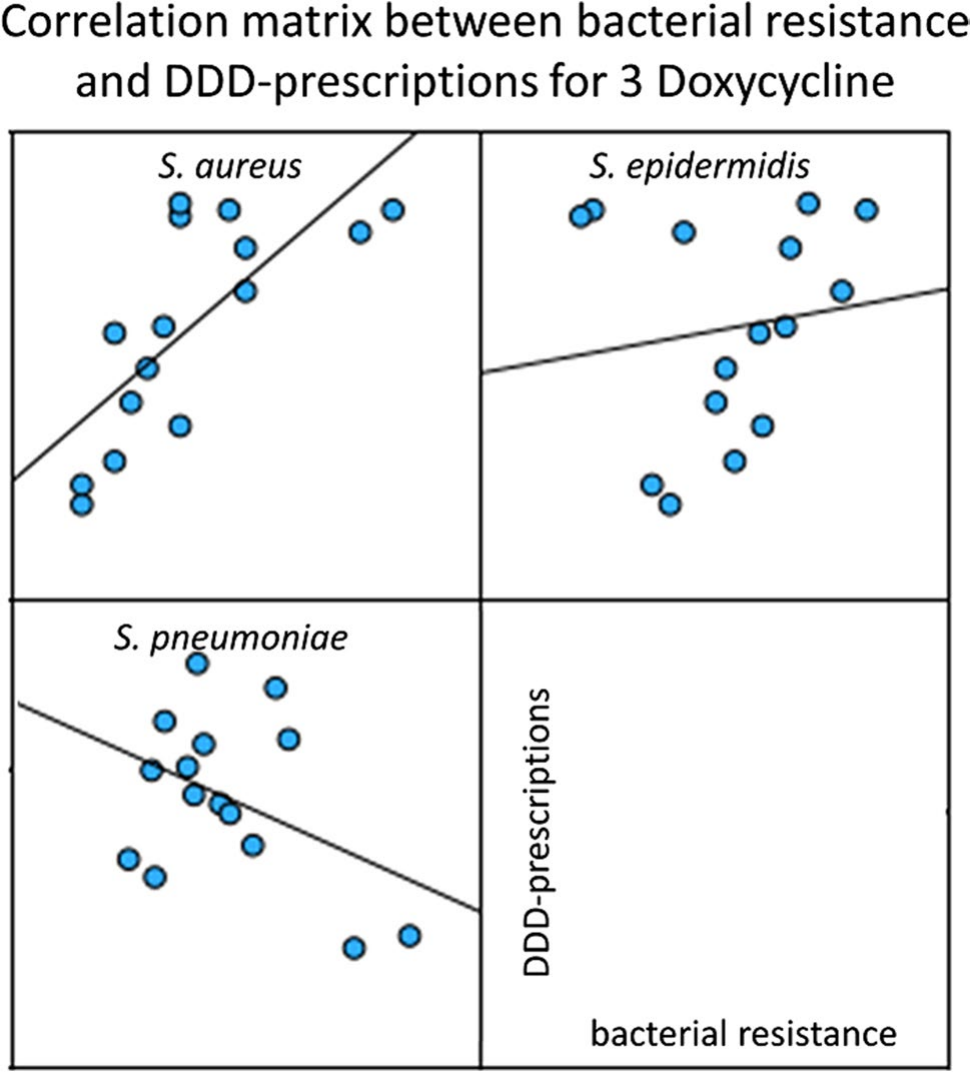
Correlation panels for the correlations between bacterial resistance and DDD-prescriptions for doxycycline. For the years 2008 to 2022, the development of bacterial resistance is plotted on the *x*-axis and DDD-prescriptions are plotted on the *y*-axis

Doxycycline, a tetracycline, was the third most prescribed antibacterial drug in 2022 (Ludwig et al. 2024). The bacterial resistance data was provided for the three pathogens *S. aureus*, *S. epidermidis*, and *S. pneumoniae*. Data can be found in Table S3 as well as a graphical visualization in Fig. 5.

Significant positive correlations for bacterial resistance vs. DDD-prescriptions were observed with *S. aureus* (0.737) and *S. pneumoniae* (0.603), although neither was strong.

### Correlation of bacterial resistance and DDD-prescriptions for amoxicillin clavulanic acid

**Fig. 6:**
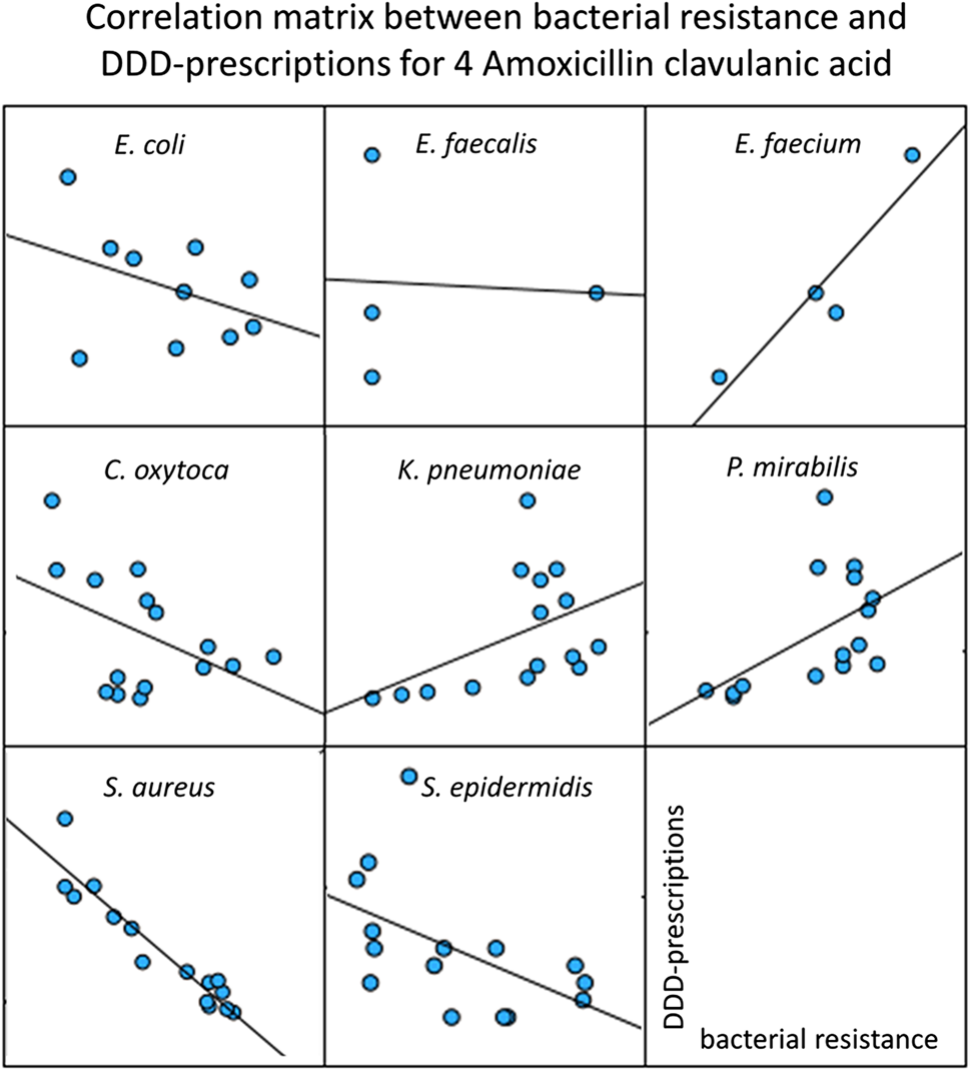
Correlation panels for the correlations between bacterial resistance and DDD-prescriptions for amoxicillin clavulanic acid. For the years 2008 to 2022, the development of bacterial resistance is plotted on the *x*-axis and DDD-prescriptions are plotted on the *y*-axis

Amoxicillin clavulanic acid, another aminopenicillin, ranked fourth in prescriptions in 2022 (Ludwig et al. 2024). Resistance data included nine pathogens: *E. coli*, *E. faecalis*, *E. faecium*, *C. oxytoca*, *K. pneumoniae*, *P. mirabilis*, *S. aureus*, *S. epidermidis*, and *S. pneumoniae*. Data can be found in Table S4 as well as a graphical visualization in Fig. 6.

Bacterial resistance and DDD-prescriptions are significantly correlated in four of nine pathogens. They divide into two positive correlations for *P. mirabilis* (0.528) and *S. pneumoniae* (0.762), and into two negative correlations for *S. aureus* (− 0.946) and *S. epidermidis* (− 0.761). The negative correlation with *S. aureus* (− 0.946) is strong.

### Correlation of bacterial resistance and DDD-prescriptions for clindamycin

**Fig. 7:**
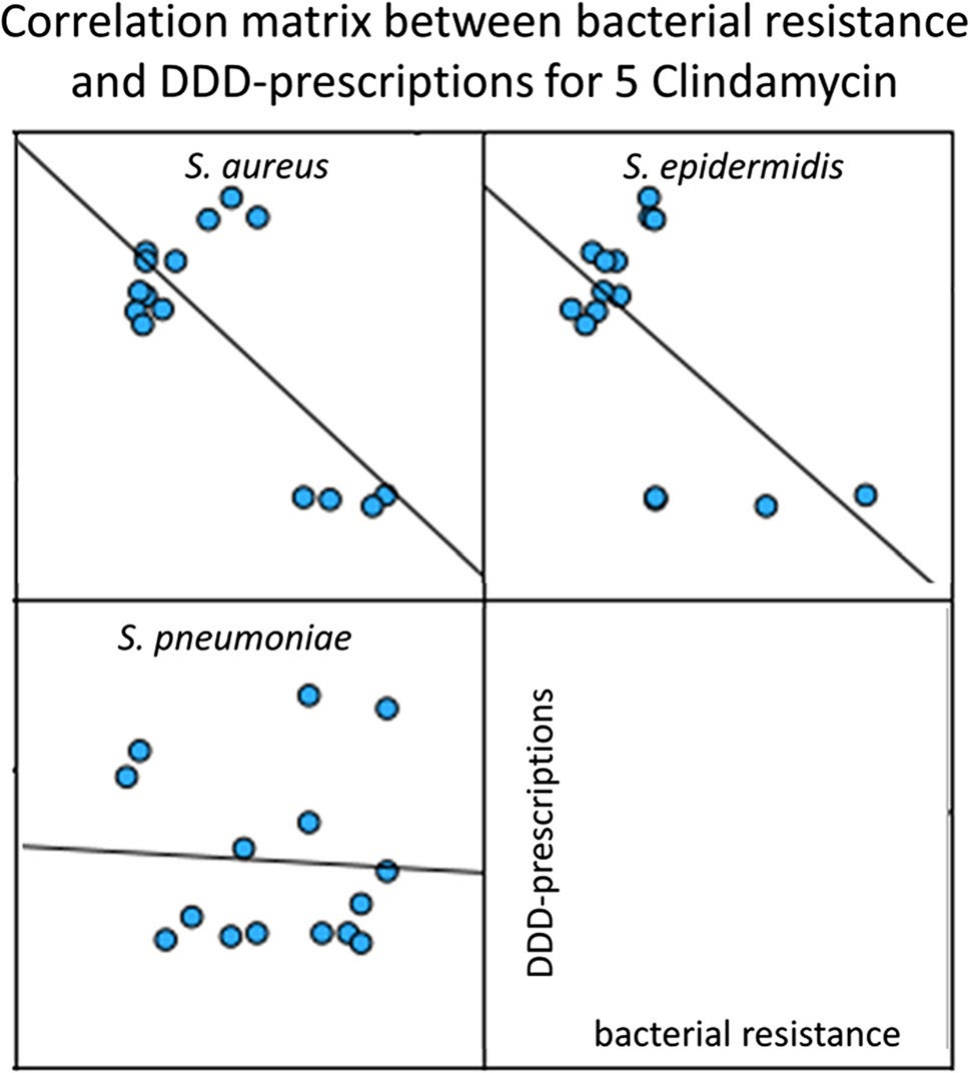
Correlation panels for the correlations between bacterial resistance and DDD-prescriptions for clindamycin. For the years 2008 to 2022, the development of bacterial resistance is plotted on the *x*-axis and DDD-prescriptions are plotted on the *y*-axis

Clindamycin is a lincosamide and is the fifth most prescribed antibacterial drug in 2022 (Ludwig et al. 2024). The bacterial resistance data includes results for the three pathogens *S. aureus*, *S. epidermidis*, and *S. pneumoniae*. Data can be found in Table S5 as well as a graphical visualization in Fig. 7.

For bacterial resistance and DDD-prescriptions, one non-significant and two significantly negative correlations are depicted, although none of them is considered as strong. The negative correlations exist with *S. aureus* (− 0.745) and *S. epidermidis* (− 0.607).

### Correlation of bacterial resistance and DDD-prescriptions for the TOP6–10 (azithromycin, sulfamethoxazole-trimethoprim, nitrofurantoin, ciprofloxacin, clarithromycin)

For reasons of conciseness, the main section of this paper focuses on the TOP5 most prescribed antibacterial drugs. The following section provides an overview analysis of the TOP6–10, which consist of azithromycin, sulfamethoxazole-trimethoprim, nitrofurantoin, ciprofloxacin, and clarithromycin. A detailed analysis can be found in the supplement. Data can be found in Table S6–10 as well as graphical visualizations in Fig. S1–S5.

Correlations between bacterial resistance and DDD-prescriptions differ in number and strength of significant pathogens. All five antibacterial drugs exhibit non-significant correlations; azithromycin has no significant correlation at all. For the other four drugs, significant correlations are depicted. Sulfamethoxazole-Trimethoprim has three significant correlations with *E. coli* (0.627), *K. pneumoniae* (− 0.688), and *E. cloacae* (–0.800). For nitrofurantoin, only one significant correlation is available with *E. coli* (0.557). Ciprofloxacin has six significant correlations, which are depicted for *E. coli* (0.922), *C. oxytoca* (0.811), *S. aureus* (0.858), *S. epidermidis* (0.817), *A. baumanii* (0.690), and *E. cloacae* (0.731). Clarithromycin has two significant correlations with *S. aureus* (0.752) and *S. epidermidis* (0.733). Strong correlations are available for sulfamethoxazole-trimethoprim with one and ciprofloxacin with four.

## Discussion

### Correlation of bacterial resistance and DDD-prescriptions

Physicians’ prescribing behaviors can be categorized as rational, semi-rational, or irrational (see Fig. 1). We hypothesized that a irrational prescribing behavior dominates in the TOP10 (Bindel and Seifert 2024b). The classification of rational, semi-rational, and irrational presription behavior is characterized by contrasting relationships between the development of bacterial resistance and DDD-prescriptions. A negative correlation between these variables suggests rational or semi-rational prescribing behavior, as both approaches consider bacterial resistance in decision-making. In rational prescribing, an increase in bacterial resistance would lead to the exclusion of the respective antibacterial drug, as treatment decisions are guided by specific pathogen information, resulting in a strong feedback loop. Semi-rational prescribing also takes resistance into account, though the feedback may be less immediate due to reliance on broader data sources and delayed analysis. Conversely, a positive correlation indicates irrational prescribing behavior, where bacterial resistance is not a factor in the selection process, and decisions are primarily driven by cost considerations, leading to the continued use of antibacterial drugs with increasing bacterial resistance.

The correlation between bacterial resistance and DDD-prescriptions can be initially characterized by examining the number of significant and strong correlations. Given the wide variability in the number of pathogens and thus the number of possible correlations, comparability can be achieved by calculating proportions. The strength of influence is measured by the share of strong versus total correlations, while the range of influences is determined by the proportion of significant versus total correlations. Tables 1 and 2 summarize the analyzed aspects.

Doxycycline, clindamycin, and clarithromycin exhibit the highest share of significant correlations among total pathogens at 66.7%. Following at a considerable interval are amoxicillin clavulanic acid (44.4%), ciprofloxacin (42.9%), and amoxicillin (40.0%). Cefuroxime axetil (28.6%) and nitrofurantoin (20.0%) show relatively low shares. Azithromycin demonstrates a share of 0.0% due to the non-significance of all analyzed pathogens. A larger influence of bacterial resistance on DDD-prescriptions corresponds to a greater number of significant correlations among the analyzed pathogens. Conversely, a lower proportion suggests a minor influence of bacterial resistance on DDD-prescriptions for most pathogens.

When examining the proportion of strong correlations among total correlations, ciprofloxacin shows the highest share at 28.6%, followed by amoxicillin clavulanic acid and sulfamethoxazole-trimethoprim at 11.1%. Other antibacterial drugs do not exhibit strong correlations, resulting in a share of 0.0%. A higher share indicates a stronger influence of bacterial resistance on DDD-prescriptions, with antibacterial drugs affected by multiple pathogens. A lower share implies limited influence by few pathogens, while a zero share indicates no strong influence by any pathogen.

There is little indication for the presence of rational prescribing behavior. Only clindamycin exhibits consistently negative correlations (see Table S5), although these may be a result of a distortion in DDD-prescriptions in 2012 (Bindel and Seifert 2024a). A comparison of the developments of bacterial resistance and DDD-prescriptions suggests that the real correlations might be positive. For azithromycin, no assessment is possible as all correlations are non-significant (see Table S6).

Semi-rational prescribing behavior is apparent for amoxicillin clavulanic acid, since both positive and negative correlations are observed in a balanced ratio (see Tables 2 and 4). Sulfamethoxazole-Trimethoprim also have both positive and negative correlations including a strong negative correlation. These data point to semirational prescribing of sulfamethoxazole-trimethoprim.

For most prescribed antibacterial drugs, a irrational prescribing behavior is present. This includes amoxicilline, cefuroxime axetil, doxycycline, nitrofurantoin, ciprofloxacin, and clarithromycin, since they only exhibits positive correlations (see Tables 2, 3, 4, S8, S9, and S10). For no antibacterial drug, we obtained evidence for rational drug prescribing.

**Table 2:** Summary of aspects regarding the analysis of the relationship between bacterial resistance and DDD-prescriptions for each antibacterial drug. In the category of significant correlations, the total number is given as the first value. The number of values that are considered to be strongly correlated, with a coefficient above (+ / −) 0.8, is given in parentheses

Ranking	Antibacterial drug	Number of analyzed pathogens	Number of significant correlations	Number of strong correlations	Number of significant positive correlations (strong positive)	Number of significant negative correlations (strong negative)	Significant correlations vs. total correlations, share in %	Strong correlations vs. total correlations, share in %	Number of pathogens with a trend of rising bacterial resistance (significant correlation)
1	Amoxicillin	5	2	0	1 (0)	1 (0)	40.0%	0.0%	4 (1)
2	Cefuroxime axetil	7	2	0	2 (0)	0	28.6%	0.0%	6 (2)
3	Doxycycline	3	2	0	2 (0)	0	66.7%	0.0%	1 (0)
4	Amoxicillin clavulanic acid	9	4	1	2 (0)	2 (1)	44.4%	11.1%	6 (2)
5	Clindamycin	3	2	0	0	2 (0)	66.7%	0.0%	0
6	Azithromycin	3	0	0	0	0	0.0%	0.0%	0
7	Sulfamethoxazole-Trimethoprim	9	3	1	1 (0)	2 (1)	33.3%	11.1%	6 (2)
8	Nitrofurantoin	5	1	0	1 (0)	0	20.0%	0.0%	4 (0)
9	Ciprofloxacin	14	6	4	6 (4)	0	42.9%	28.6%	4 (0)
10	Clarithromycin	3	2	0	2 (0)	0	66.7%	0.0%	0

**Table 3:** Summary of aspects regarding outstanding antibacterial drugs and pathogens with regard to the analysis of the correlation between bacterial resistance vs. DDD-prescriptions. In the category of significant correlations, the total number is given as the first value. The number of values that are considered to be strongly correlated, with a coefficient above (+ / −) 0.8, is given in parentheses

Antibacterial drug/pathogen	Number of analyzed pathogens/antibacterial drugs	Number of significant correlations	Number of strong correlations	Number of significant positive correlations (strong positive)	Number of significant negative correlations (strong negative)	Outstanding in number of negative/positive correlations
Sulfamethoxazole-Trimethoprim	9	3	1	1 (0)	2 (1)	Negative
Amoxicillin clavulanic acid	9	4	1	2 (0)	2 (1)	Negative
Ciprofloxacin	14	6	4	6 (4)	0	Positive

**Table 4:** Summary of mean values for bacterial resistance and DDD-costs influencing DDD-prescribing and approach to characterize prescribing behavior. For bacterial resistance, only significant correlations are included, with the number of pathogens analyzed indicating the entries used to calculate the mean. The correlations for DDD-costs (Bindel and Seifert 2024b) are derived from the same prescription data, based on the AVR. Both parameters cover the years 2008–2022, with DDD-cost data going back to 1985. Irrational prescribing is defined as a strong negative correlation between DDD-prescriptions and DDD-costs, combined with a positive correlation of mean values for significant correlations of bacterial resistance with DDD-prescriptions. If a feature is not fully developed, the characterization becomes a suggestion. Rational prescribing is defined as non-significance between DDD-prescriptions and DDD-costs and, combined with a negative correlation of mean values for significant correlations of bacterial resistance with DDD-prescriptions. Semi-rational prescribing is defined as a non-significant correlation between DDD-prescriptions and DDD-costs, combined with a negative correlation between bacterial resistance and DDD-prescriptions. If a parameter is not significant or if there are strong biases, no characterisation can be made

Ranking	Antibacterial drug	Number of significant correlations in pathogens	Average values for significant correlations with bacterial resistance (2008–2022)	Significant correlations of DDD-prescriptions and DDD-costs (1985–2022)	Characterisation of prescription behavior
**1**	Amoxicillin	2	0.008	− 0.941	Irrational
**2**	Cefuroxime axetil	2	0.563	− 0.900	Irrational
**3**	Doxycycline	2	0.670	-	?
**4**	Amoxicillin clavulanic acid	4	− 0.104	− 0.749	Semi-rational
**5**	Clindamycin	2	− 0.676	− 0.800	?
**6**	Azithromycin	0	–-	− 0.719	?
**7**	Sulfamethoxazole-Trimethoprim	3	− 0.280	− 0.758	Semi-rational
**8**	Nitrofurantoin	1	0.557	− 0.895	Irrational
**9**	Ciprofloxacin	6	0.805	− 0.533	Suggesting irrational
**10**	Clarithromycin	2	0.743	− 0.396	Suggesting irrational
	Total		0.254	− 0.743	Suggesting irrational

### Comparison of the influence of DDD-costs vs. bacterial resistance on DDD-prescriptions

To categorize the results and assess the strength of bacterial resistance’s influence on DDD-prescriptions, it is necessary to compare these results with other influential factors. DDD-costs are particularly suitable for this comparison, as they have been demonstrated to significantly impact DDD-prescriptions (Bindel and Seifert 2024b). By juxtaposing these factors, we can better understand the relative contributions of economic and microbiological considerations to prescribing behaviors for antibacterial drugs.

To facilitate a meaningful comparison, we calculated the mean value for each antibacterial drug. Given that both analyses are based on the same prescription data, this comparison is appropriate. Correlation with the longest available period was employed to minimize the effect of bias or short-term fluctuations. Table 4 presents the mean values obtained from the subsequent analysis.

The number of significant results is in both cases nine out of ten. However, the specific antibacterial drugs showing significant values differ between the two parameters. Notably, no significant average value for the correlations of bacterial resistances was observed for azithromycin, whereas doxycycline lacked significant values concerning DDD-costs (Bindel and Seifert 2024b).

The direction of correlation for each parameter also exhibits distinct patterns. Bacterial resistances predominantly show a positive correlation, with six positive and two negative correlations. Conversely, all correlations related to DDD-costs are negative.

Considering the number of strong correlations, defined by a correlation coefficient exceeding (+ / −) 0.8, there are notable differences. For bacterial resistance, ciprofloxacin is the only antibacterial drug showing a strong average correlation (see Table 1). In contrast, DDD-costs demonstrate four strong correlations, including amoxicillin, cefuroxime axetil, clindamycin, and nitrofurantoin (see Table 4). While the strong correlation is negative in DDD-costs, it is positive in bacterial resistances.

When comparing the magnitude of the parameters for each antibacterial drug, significant differences are apparent. In most cases, one parameter exhibits a strong influence while the other remains relatively minor (see Table 4). Typically, the abolute value of DDD-costs exceeds that of bacterial resistance. This includes amoxicillin, cefuroxime axetil, amoxicillin-clavulanic acid, clindamycin, sulfamethoxazole-trimethoprim, and nitrofurantoin. Conversely, for ciprofloxacin and clarithromycin, bacterial resistance has a greater impact. Due to the lack of one parameter, no assessment could be made for doxycycline and azithromycin. Summing the total of all significant values in each category reveals that DDD-prescriptions influenced by DDD-costs (− 0.743) exceed those influenced by bacterial resistances (0.254).

The reliability of the values for both parameters is high, given the availability of significant values for nearly all antibacterial drugs. Consequently, both factors can be considered stable and meaningful, allowing to draw conclusions. It is noteworthy that these parameters affect DDD-prescriptions in opposite directions. While bacterial resistance tends to develop in a manner similar to DDD-prescriptions, DDD-costs exhibit an inverse relationship. During the analyzed period, declining DDD-costs led to an increase in DDD-prescriptions, whereas bacterial resistance and DDD-prescriptions generally rose or declined similarly.

Thus, DDD-costs have a more substantial influence on DDD-prescriptions in outpatient settings than bacterial resistance in most cases. This suggests that the prescription of antibacterial drugs is primarily driven by economic considerations rather than rational and scientific facts. Although this prescribing behavior reduces health system costs, it undermines the efficacy of antibacterial drug use and promotes the development of bacterial resistance. Given the extended analysis period, this phenomenon has persisted for decades and is not merely a recent development.

### Limitations

The analysis in this study is based on data from the Arzneiverordnungsreport. As only outpatient prescriptions of the GKV system are included, no assessment can be made regarding prescriptions in hospitals or via private health insurance. As the data are based on developments in Germany, they are not directly transferable to other countries. No differentiation was made according to age or region.

Data collection for DDD-prescriptions and -costs depended on the design of the chapter under consideration. Changes in the structure of the sections over the years led to a bias for clindamycin in 2012 (Schwabe and Paffrath 2013). The ARS criteria for analyzing only indicated pathogens could exclude strong correlations, as a high increase in bacterial resistance might lead to the exclusion of the antibacterial drug in question. Both factors could lead to an under- or overestimation of the correlations in question.

Generalizations are restricted by the limited number of examined antibacterial drugs, pathogens, and factors considered. Data on bacterial resistance are only available for a short period of time compared to the available data on DDD-prescriptions and -costs. Developments in bacterial resistance prior to 2008 was not analyzed, which may lead to less accurate correlations. There may be other factors influencing the outcomes studied that were not included in the analysis.

Certain criteria were set for the statistical methods used. Between the choice of statistical procedures, the value for a significant correlation with a 2-sided significant level of 0.01 and 0.05, as well as a determined strong correlation coefficient above (+ / −) 0.8, was determined. Changing these parameters may lead to different conclusions.

## Conclusions and further perspectives

Irrational prescribing behavior predominates in the TOP10 antibacterial drugs in Germany in the outpatient setting. Irrational prescribing behavior is suggested for amoxicillin, cefuroxime axetil, doxycycline, nitrofurantoin, ciprofloxacin, and clarithromycin. For amoxicillin clavulanic acid and sulfamethoxazole-trimethoprim, hints for semi-rational prescribing behavious are evident (see Tables 2, 3, and 4). We did not identify a single drug with convincing evidence for rational prescribing. Literature research confirms our conclusions on irrational use of antibacterial drugs (Machowska et al. 2018; Sweileh 2021).

Our data also show that economic considerations dominate scientific rationale in prescribing decisions. While this may reduce healthcare costs at first sight, it raises concerns about the long-term efficacy of antibacterial therapies and the development of resistance. Given that this trend has persisted over decades, a change in prescribing practices is urgently needed to ensure the effective and safe treatment of bacterial infections in the future. There are several approaches to this. First, teaching in medical schools on proper prescribing of antibacterial drugs must improve. Second, postgraduate education of physicians on this topic must improve. Third, all stakeholders in the German health system must learn that in the long run, low DDD-costs may set wrong incentives for physicians and result in long-term health problems. Although costing time and effort, antibacteriograms should become the standard instrument for making rational decisions on antibacterial drug prescription in outpatient settings. In the long run, bacterial resistance will decrease, fewer antibacterial drugs are prescribed, fewer adverse antibacterial drug effects emerge, and overall costs for antibacterial drugs will decrease. Last but not least, the pressure to develop novel antibacterial drugs will diminish and even in decades, we can rely on established antibacterial drugs.

Further research is needed to confirm and generalize the effects of bacterial resistance and costs on DDD-prescriptions. This includes analyzing additional years and a wider range of antibacterial drugs. To determine whether irrational prescribing behavior is unique in Germany or prevalent in other countries, further comparative studies across different countries are needed. In addition, the investigation of other drug classes could show whether similar prescribing behavior occurs beyond antibacterial drugs.

## Supplementary Information

Below is the link to the electronic supplementary material.

Supplementary file1 (DOCX 1.03 MB)

## Data Availability

All source data for this study are available upon reasonable request from the authors.

## Abbreviations

AVR: Arzneiverordnungsreport (drug prescription report regarding the GKV system of Germany)
ARS: Antibiotika-Resistenz-Surveillance (surveillance of the development of bacterial resistance in antibacterial drugs)
DDD: Defined daily dose
GKV: Gesetzliche Krankenversicherung (statutory health insurance)
RKI: Robert Koch Institute
SPSS: Statistical Product and Service Solutions (software for statistical analyses, IBM)
TOP15: Group of the 15 most prescribed antibacterial drugs (based on the year 2022)
TOP10: Group of the 10 most prescribed antibacterial drugs (based on the year 2022)
TOP5: Group of the 5 most prescribed antibacterial drugs (based on the year 2022)
