# Supplementary material for: Daily defined dose-costs have a stronger influence on antibacterial drug prescriptions in Germany than bacterial resistance: economic factors are more important than scientific evidence
Source: Naunyn Schmiedebergs Arch Pharmacol. 2024 Sep 20;398(3):2909–21. doi: 10.1007/s00210-024-03435-7 (PMC11920358; doi:10.1007/s00210-024-03435-7)
Supplement: Supplementary file 1 — Supplementary file1 (DOCX 1.03 MB) [file 210_2024_3435_MOESM1_ESM.docx]

**Daily defined dose-costs have a stronger influence on antibacterial drug prescriptions in Germany than bacterial resistance: Economic factors are more important than scientific evidence**

**Lilly Josephine Bindel and Roland Seifert**

Supplemental Results

**Correlation of bacterial resistance and DDD-prescriptions for azithromycin**

Azithromycin belongs to the macrolides and is the sixth most prescribed antibacterial drug in 2022 (Ludwig et al. 2024). The bacterial resistance data includes results for *S. aureus. S. epidermidis* and *S. pneumoniae*. Data can be found in Table S6 as well as a graphical viusualisation in Fig. S1.

Regarding the correlation between bacterial resistance and DDD-prescriptions, there is no significant correlation.

**Correlation of bacterial resistance and DDD-prescriptions for sulfamethoxazole-trimethoprim**

Sulfamethoxazole-trimethoprim belongs to the substance group of sulfonamides and is the seventh most prescribed antibacterial drug in 2022 (Ludwig et al. 2024). The bacterial resistance data was available for *E. coli, C. freundii, E. cloacae, C. oxytoca, K. pneumoniae, M. morganii, P. aeruginosa, S. aureus* and *S. epidermidis*. Data can be found in Table S7 as well as a graphical viusualisation in Fig. S2.

Regarding the correlation between bacterial resistance and DDD-prescriptions, there are three significant correlations, which divide into one positive, two negative and one strongly negative correlation. They are depicted for *E. coli* (**0.627**), *K. pneumoniae* (**-0.688**), and the strong correlation for *E. cloacae* (**-0.800**).

**Correlation of bacterial resistance and DDD-prescriptions for nitrofurantoin**

Nitrofurantoin belongs to the group of “other anti-infective chemotherapeutics” and is the ninth most prescribed antibacterial drug in 2022 (Ludwig et al. 2024). Since there was no data provided for phenoxymethylpenicillin, which originally ranked before nitrofurantoin, it became ranked as number 8. The bacterial resistance data includes results for *E. coli, E. faecalis, P. mirabilis, S. aureus* and *S. epidermidis*. Data can be found in Table S8 as well as a graphical viusualisation in Fig. S3.

The bacterial resistance has a positive correlation with the DDD-prescriptions in *E. coli* (**0.557**). All other pathogens have a non-significant correlation between bacterial resistance and DDD-prescriptions.

**Correlation of bacterial resistance and DDD-prescriptions for ciprofloxacin**

Ciprofloxacin is a fluoroquinolone and the tenth most prescribed antibacterial drug in 2022 (Ludwig et al. 2024), but ranked as number 9 here. The bacterial resistance data included *E. coli, A. baumanii, C. freundii, E. cloacae, E. faecalis, E. faecium, C. oxytoca, K. pneumoniae, M. morganii, P. aeruginosa, S. marcescens, S. aureus* and *S. epidermidis*. Data can be found in Table S9 as well as a graphical viusualisation in Fig. S4.

Several significantly positive correlations between bacterial resistance and DDD-prescriptions are available, including six of fourteen pathogens. Beside this, non-significant and strong correlations are depicted too. All negative correlations are non-significant. Strong positive correlations are depicted for *E. coli* (**0.922**), *C. oxytoca* (**0.811**), *S. aureus* (**0.858**) and *S. epidermidis* (**0.817**). A significant, but not strong correlation is exhibited for *A. baumanii* (**0.690**) and *E. cloacae* (**0.731**).

**Correlation of bacterial resistance and DDD-prescriptions for clarithromycin**

Clarithromycin belongs to the substance group of macrolides and is the eleventh most prescribed antibacterial drug in 2022 (Ludwig et al. 2024), but ranked as no. 10 here. The bacterial resistance data includes results for *S. aureus*, S. *epidermidis* and *S. pneumoniae*. Data can be found in Table S10 as well as a graphical viusualisation in Fig. S5.

Regarding the correlation between bacterial resistance and DDD-prescriptions, there is a positive correlation with *S. aureus* (**0.752**) and *S. epidermidis* (**0.733**).

Supplemental Tables

***Table S1:*** *Correlation matrix, generated by SPSS, of the bacterial resistance and DDD-prescriptions for amoxicillin from 2008-2022. Dark green colour and “**” indicates a significant correlation at the 0.01 level. Light green colour and “*” indicates a significant correlation at the 0.05 level. Orange colour indicates no significant correlation.*

|  | | 1 Amoxicillin DDD-prescriptions | *E. coli*  bacterial resistance | *E. faecalis* bacterial resistance | *E. faecium* bacterial resistance | *P. mirabilis* bacterial resistance | *S. pneumoniae* bacterial resistance |
| --- | --- | --- | --- | --- | --- | --- | --- |
| 1 Amoxicillin  DDD-prescriptions | Pearson Correlation | -- |  |  |  |  |  |
|  | N | 15 |  |  |  |  |  |
| *E. coli*  bacterial resistance | Pearson Correlation | 0.679^**^ | -- |  |  |  |  |
|  | Sig. (2-tailed) | 0.005 |  |  |  |  |  |
|  | R^2^ | 0.461 |  |  |  |  |  |
|  | N | 15 | 15 |  |  |  |  |
| *E. faecalis*  bacterial resistance | Pearson Correlation | -0.546 | -0.352 | -- |  |  |  |
|  | Sig. (2-tailed) | 0.161 | 0.392 |  |  |  |  |
|  | R^2^ | 0.298 | 0.124 |  |  |  |  |
|  | N | 8 | 8 | 8 |  |  |  |
| *E. faecium*  bacterial resistance | Pearson Correlation | 0.694 | -1.000^*^ | 0.017 | -- |  |  |
|  | Sig. (2-tailed) | 0.511 | 0.011 | 0.989 |  |  |  |
|  | R^2^ | 0.481 | 1.000 | 0.000 |  |  |  |
|  | N | 3 | 3 | 3 | 3 |  |  |
| *P. mirabilis*  bacterial resistance | Pearson Correlation | 0.417 | 0.724^**^ | -0.265 | -0.875 | -- |  |
|  | Sig. (2-tailed) | 0.122 | 0.002 | 0.527 | 0.322 |  |  |
|  | R^2^ | 0.174 | 0.524 | 0.070 | 0.766 |  |  |
|  | N | 15 | 15 | 8 | 3 | 15 |  |
| *S. pneumoniae*  bacterial resistance | Pearson Correlation | -0.663^**^ | -0.491 | -0.031 | -0.413 | -0.036 | -- |
|  | Sig. (2-tailed) | 0.007 | 0.063 | 0.943 | 0.729 | 0.898 |  |
|  | R^2^ | 0.440 | 0.241 | 0.001 | 0.171 | 0.001 |  |
|  | N | 15 | 15 | 8 | 3 | 15 | 15 |

***Table S2:*** *Correlation matrix, generated by SPSS, of the bacterial resistance and DDD-prescriptions for cefuroxime axetil from 2008-2022. Dark green colour and “**” indicates a significant correlation at the 0.01 level. Light green colour and “*” indicates a significant correlation at the 0.05 level. Orange colour indicates no significant correlation.*

|  | | 2 Cefuroxime axetil DDD-prescriptions | *E. coli* bacterial resistance | *C. oxytoca* bacterial resistance | *K. pneumoniae* bacterial resistance | *P. mirabilis* bacterial resistance | *S. aureus* bacterial resistance | *S. epidermidis* bacterial resistance | *S. pneumoniae* bacterial resistance |
| --- | --- | --- | --- | --- | --- | --- | --- | --- | --- |
| 2 Cefuroxime axetil DDD-prescriptions | Pearson Correlation | -- |  |  |  |  |  |  |  |
|  | N | 33 |  |  |  |  |  |  |  |
| *E. coli*  bacterial resistance | Pearson Correlation | 0.570^*^ | -- |  |  |  |  |  |  |
|  | Sig. (2-tailed) | 0.026 |  |  |  |  |  |  |  |
|  | R^2^ | 0.325 |  |  |  |  |  |  |  |
|  | N | 15 | 15 |  |  |  |  |  |  |
| *C. oxytoca*  bacterial resistance | Pearson Correlation | -0.045 | -0.595^*^ | -- |  |  |  |  |  |
|  | Sig. (2-tailed) | 0.872 | 0.019 |  |  |  |  |  |  |
|  | R^2^ | 0.002 | 0.354 |  |  |  |  |  |  |
|  | N | 15 | 15 | 15 |  |  |  |  |  |
| *K. pneumoniae*  bacterial resistance | Pearson Correlation | 0.555^*^ | 0.962^**^ | -0.427 | -- |  |  |  |  |
|  | Sig. (2-tailed) | 0.032 | 0.000 | 0.113 |  |  |  |  |  |
|  | R^2^ | 0.308 | 0.925 | 0.182 |  |  |  |  |  |
|  | N | 15 | 15 | 15 | 15 |  |  |  |  |
| *P. mirabilis*  bacterial resistance | Pearson Correlation | 0.088 | 0.726^**^ | -0.382 | 0.794^**^ | -- |  |  |  |
|  | Sig. (2-tailed) | 0.755 | 0.002 | 0.160 | 0.000 |  |  |  |  |
|  | R^2^ | 0.001 | 0.527 | 0.146 | 0.630 |  |  |  |  |
|  | N | 15 | 15 | 15 | 15 | 15 |  |  |  |
| *S. aureus*  bacterial resistance | Pearson Correlation | -0.001 | -0.777^**^ | 0.674^**^ | -0.734^**^ | -0.799^**^ | -- |  |  |
|  | Sig. (2-tailed) | 0.997 | 0.001 | 0.006 | 0.002 | 0.000 |  |  |  |
|  | R^2^ | 0.000 | 0.604 | 0.454 | 0.539 | 0.638 |  |  |  |
|  | N | 15 | 15 | 15 | 15 | 15 | 15 |  |  |
| *S. epidermidis*  bacterial resistance | Pearson Correlation | 0.455 | 0.071 | 0.577^*^ | 0.206 | -0.011 | 0.234 | -- |  |
|  | Sig. (2-tailed) | 0.088 | 0.802 | 0.024 | 0.462 | 0.970 | 0.401 |  |  |
|  | R^2^ | 0.207 | 0.005 | 0.333 | 0.424 | 0.000 | 0.055 |  |  |
|  | N | 15 | 15 | 15 | 15 | 15 | 15 | 15 |  |
| *S. pneumoniae*  bacterial resistance | Pearson Correlation | -0.230 | 0.561^*^ | -0.371 | 0.588^*^ | 0.842^**^ | -0.846^**^ | -0.059 | -- |
|  | Sig. (2-tailed) | 0.410 | 0.030 | 0.174 | 0.021 | 0.000 | 0.000 | 0.835 |  |
|  | R^2^ | 0.053 | 0.315 | 0.138 | 0.346 | 0.709 | 0.716 | 0.003 |  |
|  | N | 15 | 15 | 15 | 15 | 15 | 15 | 15 | 15 |

***Table S3:*** *Correlation matrix, generated by SPSS, of the bacterial resistance and DDD-prescriptions for doxycycline from 2008-2022. Dark green colour and “**” indicates a significant correlation at the 0.01 level. Light green colour and “*” indicates a significant correlation at the 0.05 level. Orange colour indicates no significant correlation.*

|  | | 3 Doxycycline DDD-prescriptions | *S. aureus*  bacterial resistance | *S. epidermidis* bacterial resistance | *S. pneumoniae* bacterial resistance |
| --- | --- | --- | --- | --- | --- |
| 3 Doxycycline  DDD-prescriptions | Pearson Correlation | -- |  |  |  |
|  | N | 38 |  |  |  |
| *S. aureus*  bacterial resistance | Pearson Correlation | 0.737^**^ | -- |  |  |
|  | Sig. (2-tailed) | 0.002 |  |  |  |
|  | R^2^ | 0.543 |  |  |  |
|  | N | 15 | 15 |  |  |
| *S. epidermidis*  bacterial resistance | Pearson Correlation | 0.140 | 0.335 | -- |  |
|  | Sig. (2-tailed) | 0.619 | 0.222 |  |  |
|  | R^2^ | 0.019 | 0.112 |  |  |
|  | N | 15 | 15 | 15 |  |
| *S. pneumoniae*  bacterial resistance | Pearson Correlation | 0.603^*^ | 0.386 | -0.418 | -- |
|  | Sig. (2-tailed) | 0.017 | 0.156 | 0.121 |  |
|  | R^2^ | 0.364 | 0.149 | 0.175 |  |
|  | N | 15 | 15 | 15 | 15 |

***Table S4:*** *Correlation matrix, generated by SPSS, of the bacterial resistance and DDD-prescriptions for amoxicillin clavulanic acid from 2008-2022. Dark green colour and “**” indicates a significant correlation at the 0.01 level. Light green colour and “*” indicates a significant correlation at the 0.05 level. Orange colour indicates no significant correlation.*

|  | | 4 Amoxicillin clavulanic acid DDD-prescriptions | *E. coli* bacterial resistance | *E. faecalis* bacterial resistance | *E. faecium* bacterial resistance | *C. oxytoca* bacterial resistance | *K. pneumoniae* bacterial resistance | *P. mirabilis* bacterial resistance | *S. aureus* bacterial resistance | *S. epidermidis* bacterial resistance | *S. pneumoniae* bacterial resistance |
| --- | --- | --- | --- | --- | --- | --- | --- | --- | --- | --- | --- |
| 4 Amoxicillin clavulanic acid  DDD-prescriptions | Pearson Correlation | -- |  |  |  |  |  |  |  |  |  |
|  | N | 37 |  |  |  |  |  |  |  |  |  |
| *E. coli*  bacterial resistance | Pearson Correlation | 0.457 | -- |  |  |  |  |  |  |  |  |
|  | Sig. (2-tailed) | 0.086 |  |  |  |  |  |  |  |  |  |
|  | R^2^ | 0.209 |  |  |  |  |  |  |  |  |  |
|  | N | 15 | 15 |  |  |  |  |  |  |  |  |
| *E. faecalis*  bacterial resistance | Pearson Correlation | 0.365 | -0.080 | -- |  |  |  |  |  |  |  |
|  | Sig. (2-tailed) | 0.300 | 0.826 |  |  |  |  |  |  |  |  |
|  | R^2^ | 0.133 | 0.006 |  |  |  |  |  |  |  |  |
|  | N | 10 | 10 | 10 |  |  |  |  |  |  |  |
| *E. faecium*  bacterial resistance | Pearson Correlation | 0.937 | -0.955^*^ | -0.042 | -- |  |  |  |  |  |  |
|  | Sig. (2-tailed) | 0.063 | 0.045 | 0.958 |  |  |  |  |  |  |  |
|  | R^2^ | 0.878 | 0.912 | 0.002 |  |  |  |  |  |  |  |
|  | N | 4 | 4 | 4 | 4 |  |  |  |  |  |  |
| *C. oxytoca*  bacterial resistance | Pearson Correlation | -0.465 | 0.440 | -0.322 | -0.866 | -- |  |  |  |  |  |
|  | Sig. (2-tailed) | 0.080 | 0.101 | 0.364 | 0.134 |  |  |  |  |  |  |
|  | R^2^ | 0.216 | 0.194 | 0.104 | 0.750 |  |  |  |  |  |  |
|  | N | 15 | 15 | 10 | 4 | 15 |  |  |  |  |  |
| *K. pneumoniae* bacterial resistance | Pearson Correlation | 0.464 | 0.932^**^ | -0.248 | -0.469 | 0.382 | -- |  |  |  |  |
|  | Sig. (2-tailed) | 0.081 | 0.000 | 0.489 | 0.531 | 0.160 |  |  |  |  |  |
|  | R^2^ | 0.215 | 0.869 | 0.062 | 0.220 | 0.146 |  |  |  |  |  |
|  | N | 15 | 15 | 10 | 4 | 15 | 15 |  |  |  |  |
| *P. mirabilis*  bacterial resistance | Pearson Correlation | 0.528^*^ | 0.973^**^ | -0.183 | -0.688 | 0.349 | 0.896^**^ | -- |  |  |  |
|  | Sig. (2-tailed) | 0.043 | 0.000 | 0.612 | 0.312 | 0.202 | 0.000 |  |  |  |  |
|  | R^2^ | 0.279 | 0.947 | 0.335 | 0.473 | 0.122 | 0.534 |  |  |  |  |
|  | N | 15 | 15 | 10 | 4 | 15 | 15 | 15 |  |  |  |
| *S. aureus*  bacterial resistance | Pearson Correlation | -0.946^**^ | -0.443 | -0.438 | -0.867 | 0.468 | -0.475 | -0.508 | -- |  |  |
|  | Sig. (2-tailed) | 0.000 | 0.098 | 0.206 | 0.133 | 0.079 | 0.074 | 0.053 |  |  |  |
|  | R^2^ | 0.895 | 0.196 | 0.192 | 0.751 | 0.219 | 0.226 | 0.258 |  |  |  |
|  | N | 15 | 15 | 10 | 4 | 15 | 15 | 15 | 15 |  |  |
| *S. epidermidis*  bacterial resistance | Pearson Correlation | -0.761^**^ | -0.254 | -0.401 | 0.088 | 0.422 | -0.233 | -0.344 | 0.820^**^ | -- |  |
|  | Sig. (2-tailed) | 0.001 | 0.362 | 0.251 | 0.912 | 0.117 | 0.403 | 0.210 | 0.000 |  |  |
|  | R^2^ | 0.579 | 0.064 | 0.161 | 0.008 | 0.178 | 0.054 | 0.118 | 0.672 |  |  |
|  | N | 15 | 15 | 10 | 4 | 15 | 15 | 15 | 15 | 15 |  |
| *S. pneumoniae* bacterial resistance | Pearson Correlation | 0.762^**^ | 0.359 | 0.115 | 0.675 | -0.449 | 0.404 | 0.403 | -0.800^**^ | -0.524^*^ | -- |
|  | Sig. (2-tailed) | 0.001 | 0.188 | 0.751 | 0.325 | 0.093 | 0.136 | 0.137 | 0.000 | 0.045 |  |
|  | R^2^ | 0.580 | 0.129 | 0.013 | 0.456 | 0.202 | 0.163 | 0.162 | 0.640 | 0.275 |  |
|  | N | 15 | 15 | 10 | 4 | 15 | 15 | 15 | 15 | 15 | 15 |

***Table S5:*** *Correlation matrix, generated by SPSS, of the bacterial resistance and DDD-prescriptions for clindamycin from 2008-2022. Dark green colour and “**” indicates a significant correlation at the 0.01 level. Light green colour and “*” indicates a significant correlation at the 0.05 level. Orange colour indicates no significant correlation.*

|  | | 5 Clindamycin  DDD-prescriptions | *S. aureus*  bacterial resistance | *S. epidermidis* bacterial resistance | *S. pneumoniae* bacterial resistance |
| --- | --- | --- | --- | --- | --- |
| 5 Clindamycin  DDD-prescriptions | Pearson Correlation | -- |  |  |  |
|  | N | 37 |  |  |  |
| *S. aureus*  bacterial resistance | Pearson Correlation | -0.745^**^ | -- |  |  |
|  | Sig. (2-tailed) | 0.001 |  |  |  |
|  | R^2^ | 0.555 |  |  |  |
|  | N | 15 | 15 |  |  |
| *S. epidermidis*  bacterial resistance | Pearson Correlation | -0.607^*^ | 0.866^**^ | -- |  |
|  | Sig. (2-tailed) | 0.016 | 0.000 |  |  |
|  | R^2^ | 0.368 | 0.750 |  |  |
|  | N | 15 | 15 | 15 |  |
| *S. pneumoniae*  bacterial resistance | Pearson Correlation | 0.298 | -0.058 | 0.223 | -- |
|  | Sig. (2-tailed) | 0.281 | 0.839 | 0.423 |  |
|  | R^2^ | 0.088 | 0.003 | 0.497 |  |
|  | N | 15 | 15 | 15 | 15 |

***Table S6:*** *Correlation matrix, generated by SPSS, of the bacterial resistance and DDD-prescriptions for azithromycin from 2008-2022. Dark green colour and “**” indicates a significant correlation at the 0.01 level. Light green colour and “*” indicates a significant correlation at the 0.05 level. Orange colour indicates no significant correlation.*

|  | | 6 Azithromycin DDD-prescriptions | *S. aureus*  bacterial resistance | *S. epidermidis* bacterial resistance | *S. pneumoniae* bacterial resistance |
| --- | --- | --- | --- | --- | --- |
| 6 Azithromycin  DDD-prescriptions | Pearson Correlation | -- |  |  |  |
|  | N | 29 |  |  |  |
| *S. aureus*  bacterial resistance | Pearson Correlation | -0.085 | -- |  |  |
|  | Sig. (2-tailed) | 0.763 |  |  |  |
|  | R^2^ | 0.007 |  |  |  |
|  | N | 15 | 15 |  |  |
| *S. epidermidis*  bacterial resistance | Pearson Correlation | 0.341 | 0.415 | -- |  |
|  | Sig. (2-tailed) | 0.214 | 0.124 |  |  |
|  | R^2^ | 0.116 | 0.172 |  |  |
|  | N | 15 | 15 | 15 |  |
| *S. pneumoniae*  bacterial resistance | Pearson Correlation | 0.357 | -0.657 | -0.028 | -- |
|  | Sig. (2-tailed) | 0.346 | 0.054 | 0.943 |  |
|  | R^2^ | 0.127 | 0.431 | 0.001 |  |
|  | N | 9 | 9 | 9 | 9 |

***Table S7:*** *Correlation matrix, generated by SPSS, of the bacterial resistance and DDD-prescriptions for sulfamethoxazole-trimethoprim from 2008-2022. Dark green colour and “**” indicates a significant correlation at the 0.01 level. Light green colour and “*” indicates a significant correlation at the 0.05 level. Orange colour indicates no significant correlation.*

|  | | 7 Sulfamethoxazole-Trimethoprim  DDD-prescriptions | *E. coli*  bacterial resistance | *C. freundii*  bacterial resistance | *E. cloacae*  bacterial resistance | *C. oxytoca*  bacterial resistance | *K. pneumoniae* bacterial resistance | *M. morganii*  bacterial resistance | *P. aeruginosa*  bacterial resistance | *S. aureus*  bacterial resistance | *S. epidermidis*  bacterial resistance |
| --- | --- | --- | --- | --- | --- | --- | --- | --- | --- | --- | --- |
| 7 Sulfamethoxazole-Trimethoprim DDD-prescriptions | Pearson Correlation | -- |  |  |  |  |  |  |  |  |  |
|  | N | 38 |  |  |  |  |  |  |  |  |  |
| *E. coli*  bacterial resistance | Pearson Correlation | 0.627^*^ | -- |  |  |  |  |  |  |  |  |
|  | Sig. (2-tailed) | 0.012 |  |  |  |  |  |  |  |  |  |
|  | R^2^ | 0.393 |  |  |  |  |  |  |  |  |  |
|  | N | 15 | 15 |  |  |  |  |  |  |  |  |
| *C. freundii* bacterial resistance | Pearson Correlation | -0.232 | 0.067 | -- |  |  |  |  |  |  |  |
|  | Sig. (2-tailed) | 0.468 | 0.835 |  |  |  |  |  |  |  |  |
|  | R^2^ | 0.538 | 0.004 |  |  |  |  |  |  |  |  |
|  | N | 12 | 12 | 12 |  |  |  |  |  |  |  |
| *E. cloacae* bacterial resistance | Pearson Correlation | -0.800^**^ | -0.519 | 0.299 | -- |  |  |  |  |  |  |
|  | Sig. (2-tailed) | 0.002 | 0.084 | 0.345 |  |  |  |  |  |  |  |
|  | R^2^ | 0.640 | 0.269 | 0.089 |  |  |  |  |  |  |  |
|  | N | 12 | 12 | 12 | 12 |  |  |  |  |  |  |
| *C. oxytoca* bacterial resistance | Pearson Correlation | -0.341 | 0.402 | 0.329 | 0.480 | -- |  |  |  |  |  |
|  | Sig. (2-tailed) | 0.277 | 0.195 | 0.297 | 0.114 |  |  |  |  |  |  |
|  | R^2^ | 0.116 | 0.161 | 0.088 | 0.230 |  |  |  |  |  |  |
|  | N | 12 | 12 | 12 | 12 | 12 |  |  |  |  |  |
| *K. pneumoniae* bacterial resistance | Pearson Correlation | -0.668^*^ | -0.339 | 0.383 | 0.838^**^ | 0.600^*^ | -- |  |  |  |  |
|  | Sig. (2-tailed) | 0.013 | 0.257 | 0.219 | 0.001 | 0.039 |  |  |  |  |  |
|  | R^2^ | 0.446 | 0.114 | 0.147 | 0.702 | 0.360 |  |  |  |  |  |
|  | N | 13 | 13 | 12 | 12 | 12 | 13 |  |  |  |  |
| *M. morganii* bacterial resistance | Pearson Correlation | -0.367 | 0.253 | 0.088 | 0.616^*^ | 0.724^**^ | 0.582^*^ | -- |  |  |  |
|  | Sig. (2-tailed) | 0.241 | 0.427 | 0.786 | 0.033 | 0.008 | 0.047 |  |  |  |  |
|  | R^2^ | 0.135 | 0.064 | 0.008 | 0.379 | 0.524 | 0.339 |  |  |  |  |
|  | N | 12 | 12 | 12 | 12 | 12 | 12 | 12 |  |  |  |
| *P. aeruginosa* bacterial resistance | Pearson Correlation | -0.425 | -0.167 | -0.295 | 0.566 | 0.292 | 0.603^*^ | 0.728^**^ | -- |  |  |
|  | Sig. (2-tailed) | 0.169 | 0.603 | 0.352 | 0.055 | 0.357 | 0.038 | 0.007 |  |  |  |
|  | R^2^ | 0.180 | 0.028 | 0.087 | 0.320 | 0.058 | 0.364 | 0.530 |  |  |  |
|  | N | 12 | 12 | 12 | 12 | 12 | 12 | 12 | 12 |  |  |
| *S. aureus* bacterial resistance | Pearson Correlation | 0.265 | -0.882^**^ | -0.516 | -0.694 | -0.819^*^ | -0.473 | -0.795^*^ | -0.346 | -- |  |
|  | Sig. (2-tailed) | 0.527 | 0.004 | 0.191 | 0.056 | 0.013 | 0.237 | 0.018 | 0.401 |  |  |
|  | R^2^ | 0.070 | 0.778 | 0.266 | 0.481 | 0.671 | 0.224 | 0.632 | 0.119 |  |  |
|  | N | 8 | 8 | 8 | 8 | 8 | 8 | 8 | 8 | 8 |  |
| *S. epidermidis* bacterial resistance | Pearson Correlation | -0.342 | 0.993^**^ | 0.409 | 0.898^**^ | 0.974^**^ | 0.759^*^ | 0.981^**^ | 0.708^*^ | -0.860^**^ | -- |
|  | Sig. (2-tailed) | 0.408 | 0.000 | 0.314 | 0.002 | 0.000 | 0.029 | 0.000 | 0.049 | 0.006 |  |
|  | R^2^ | 0.117 | 0.986 | 0.167 | 0.806 | 0.949 | 0.576 | 0.962 | 0.501 | 0.740 |  |
|  | N | 8 | 8 | 8 | 8 | 8 | 8 | 8 | 8 | 8 | 8 |

***Table S8:*** *Correlation matrix, generated by SPSS, of the bacterial resistance and DDD-prescriptions for nitrofurantoin from 2008-2022. Dark green colour and “**” indicates a significant correlation at the 0.01 level. Light green colour and “*” indicates a significant correlation at the 0.05 level. Orange colour indicates no significant correlation.*

|  | | 8 Nitrofurantoin DDD-prescriptions | *E. coli*  bacterial resistance | *E. faecalis* bacterial resistance | *P. mirabilis* bacterial resistance | *S. aureus* bacterial resistance | *S. epidermidis* bacterial resistance |
| --- | --- | --- | --- | --- | --- | --- | --- |
| 8 Nitrofurantoin  DDD-prescriptions | Pearson Correlation | -- |  |  |  |  |  |
|  | N | 16 |  |  |  |  |  |
| *E. coli*  bacterial resistance | Pearson Correlation | 0.557^*^ | -- |  |  |  |  |
|  | Sig. (2-tailed) | 0.031 |  |  |  |  |  |
|  | R^2^ | 0.310 |  |  |  |  |  |
|  | N | 15 | 15 |  |  |  |  |
| *E. faecalis*  bacterial resistance | Pearson Correlation | -0.666 | -0.344 | -- |  |  |  |
|  | Sig. (2-tailed) | 0.050 | 0.364 |  |  |  |  |
|  | R^2^ | 0.444 | 0.118 |  |  |  |  |
|  | N | 9 | 9 | 9 |  |  |  |
| *P. mirabilis*  bacterial resistance | Pearson Correlation | -0.418 | -0.693^**^ | 0.401 | -- |  |  |
|  | Sig. (2-tailed) | 0.156 | 0.009 | 0.285 |  |  |  |
|  | R^2^ | 0.174 | 0.480 | 0.161 |  |  |  |
|  | N | 13 | 13 | 9 | 13 |  |  |
| *S. aureus*  bacterial resistance | Pearson Correlation | -0.197 | -0.830^**^ | 0.377 | 0.582^*^ | -- |  |
|  | Sig. (2-tailed) | 0.481 | 0.000 | 0.318 | 0.037 |  |  |
|  | R^2^ | 0.039 | 0.689 | 0.142 | 0.339 |  |  |
|  | N | 15 | 15 | 9 | 13 | 15 |  |
| *S. epidermidis*  bacterial resistance | Pearson Correlation | -0.187 | -0.804^**^ | 0.568 | 0.656^*^ | 0.958^**^ | -- |
|  | Sig. (2-tailed) | 0.505 | 0.000 | 0.111 | 0.015 | 0.000 |  |
|  | R^2^ | 0.035 | 0.646 | 0.323 | 0.430 | 0.918 |  |
|  | N | 15 | 15 | 9 | 13 | 15 | 15 |

***Table S9:*** *Correlation matrix, generated by SPSS, of the bacterial resistance and DDD-prescriptions for ciprofloxacin from 2008-2022. Dark green colour and “**” indicates a significant correlation at the 0.01 level. Light green colour and “*” indicates a significant correlation at the 0.05 level. Orange colour indicates no significant correlation*

|  | | 9 Ciprofloxacin DDD-prescriptions | *E. coli*  bacterial resistance | *A. baumannii* bacterial resistance | *C. freundii* bacterial resistance | *E. cloacae* bacterial resistance | *E. faecalis* bacterial resistance | *E. faecium* bacterial resistance | *C. oxytoca* bacterial resistance | *K. pneumoniae* bacterial resistance | *M. morganii* bacterial resistance | *P. mirabilis* bacterial resistance | *P. aeruginosa* bacterial resistance | *S. marcescens* bacterial resistance | *S. aureus* bacterial resistance | *S. epidermidis* bacterial resistance |
| --- | --- | --- | --- | --- | --- | --- | --- | --- | --- | --- | --- | --- | --- | --- | --- | --- |
| 9 Ciprofloxacin DDD-prescriptions | Pearson Correlation | -- |  |  |  |  |  |  |  |  |  |  |  |  |  |  |
|  | N | 36 |  |  |  |  |  |  |  |  |  |  |  |  |  |  |
| *E. coli* bacterial resistance | Pearson Correlation | 0.922^**^ | -- |  |  |  |  |  |  |  |  |  |  |  |  |  |
|  | Sig. (2-tailed) | 0.000 |  |  |  |  |  |  |  |  |  |  |  |  |  |  |
|  | R^2^ | 0.850 |  |  |  |  |  |  |  |  |  |  |  |  |  |  |
|  | N | 15 | 15 |  |  |  |  |  |  |  |  |  |  |  |  |  |
| *A. baumannii* bacterial resistance | Pearson Correlation | 0.690^**^ | 0.685^**^ | -- |  |  |  |  |  |  |  |  |  |  |  |  |
|  | Sig. (2-tailed) | 0.004 | 0.005 |  |  |  |  |  |  |  |  |  |  |  |  |  |
|  | R^2^ | 0.476 | 0.469 |  |  |  |  |  |  |  |  |  |  |  |  |  |
|  | N | 15 | 15 | 15 |  |  |  |  |  |  |  |  |  |  |  |  |
| *C. freundii* bacterial resistance | Pearson Correlation | 0.001 | 0.142 | 0.458 | -- |  |  |  |  |  |  |  |  |  |  |  |
|  | Sig. (2-tailed) | 0.997 | 0.613 | 0.086 |  |  |  |  |  |  |  |  |  |  |  |  |
|  | R^2^ | 0.000 | 0.020 | 0.209 |  |  |  |  |  |  |  |  |  |  |  |  |
|  | N | 15 | 15 | 15 | 15 |  |  |  |  |  |  |  |  |  |  |  |
| *E. cloacae* bacterial resistance | Pearson Correlation | 0.731^**^ | 0.819^**^ | 0.743^**^ | 0.287 | -- |  |  |  |  |  |  |  |  |  |  |
|  | Sig. (2-tailed) | 0.002 | 0.000 | 0.002 | 0.299 |  |  |  |  |  |  |  |  |  |  |  |
|  | R^2^ | 0.534 | 0.670 | 0.552 | 0.082 |  |  |  |  |  |  |  |  |  |  |  |
|  | N | 15 | 15 | 15 | 15 | 15 |  |  |  |  |  |  |  |  |  |  |
| *E. faecalis* bacterial resistance | Pearson Correlation | 0.019 | 0.067 | -0.435 | -0.440 | -0.064 | -- |  |  |  |  |  |  |  |  |  |
|  | Sig. (2-tailed) | 0.945 | 0.813 | 0.105 | 0.100 | 0.821 |  |  |  |  |  |  |  |  |  |  |
|  | R^2^ | 0.000 | 0.004 | 0.189 | 0.194 | 0.004 |  |  |  |  |  |  |  |  |  |  |
|  | N | 15 | 15 | 15 | 15 | 15 | 15 |  |  |  |  |  |  |  |  |  |
| *E. faecium* bacterial resistance | Pearson Correlation | -0.561 | -0.615 | -0.597 | -0.197 | -0.597 | -0.513 | -- |  |  |  |  |  |  |  |  |
|  | Sig. (2-tailed) | 0.621 | 0.578 | 0.593 | 0.874 | 0.593 | 0.657 |  |  |  |  |  |  |  |  |  |
|  | R^2^ | 0.315 | 0.378 | 0.356 | 0.039 | 0.356 | 0.263 |  |  |  |  |  |  |  |  |  |
|  | N | 3 | 3 | 3 | 3 | 3 | 3 | 3 |  |  |  |  |  |  |  |  |
| *C. oxytoca* bacterial resistance | Pearson Correlation | 0.811^**^ | 0.809^**^ | 0.945^**^ | 0.422 | 0.784^**^ | -0.281 | -0.685 | -- |  |  |  |  |  |  |  |
|  | Sig. (2-tailed) | 0.000 | 0.000 | 0.000 | 0.117 | 0.001 | 0.310 | 0.520 |  |  |  |  |  |  |  |  |
|  | R^2^ | 0.658 | 0.654 | 0.893 | 0.178 | 0.615 | 0.078 | 0.469 |  |  |  |  |  |  |  |  |
|  | N | 15 | 15 | 15 | 15 | 15 | 15 | 3 | 15 |  |  |  |  |  |  |  |
| *K. pneumoniae* bacterial resistance | Pearson Correlation | -0.151 | 0.040 | -0.546^*^ | -0.191 | -0.001 | 0.492 | -0.522 | -0.422 | -- |  |  |  |  |  |  |
|  | Sig. (2-tailed) | 0.590 | 0.888 | 0.035 | 0.495 | 0.998 | 0.062 | 0.651 | 0.117 |  |  |  |  |  |  |  |
|  | R^2^ | 0.023 | 0.002 | 0.298 | 0.036 | 0.000 | 0.242 | 0.272 | 0.178 |  |  |  |  |  |  |  |
|  | N | 15 | 15 | 15 | 15 | 15 | 15 | 3 | 15 | 15 |  |  |  |  |  |  |
| *M. morganii* bacterial resistance | Pearson Correlation | -0.484 | -0.297 | -0.786^**^ | -0.205 | -0.362 | 0.524^*^ | -0.796 | -0.691^**^ | 0.794^**^ | -- |  |  |  |  |  |
|  | Sig. (2-tailed) | 0.068 | 0.283 | 0.001 | 0.463 | 0.185 | 0.045 | 0.414 | 0.004 | 0.000 |  |  |  |  |  |  |
|  | R^2^ | 0.234 | 0.088 | 0.618 | 0.042 | 0.131 | 0.274 | 0.634 | 0.478 | 0.630 |  |  |  |  |  |  |
|  | N | 15 | 15 | 15 | 15 | 15 | 15 | 3 | 15 | 15 | 15 |  |  |  |  |  |
| *P. mirabilis* bacterial resistance | Pearson Correlation | -0.454 | -0.297 | -0.831^**^ | -0.294 | -0.385 | 0.645^**^ | -0.729 | -0.060^**^ | 0.750^**^ | 0.944^**^ | -- |  |  |  |  |
|  | Sig. (2-tailed) | 0.089 | 0.283 | 0.000 | 0.287 | 0.156 | 0.009 | 0.480 | 0.003 | 0.001 | 0.000 |  |  |  |  |  |
|  | R^2^ | 0.206 | 0.088 | 0.690 | 0.086 | 0.148 | 0.416 | 0.531 | 0.004 | 0.563 | 0.891 |  |  |  |  |  |
|  | N | 15 | 15 | 15 | 15 | 15 | 15 | 3 | 15 | 15 | 15 | 15 |  |  |  |  |
| *P. aeruginosa* bacterial resistance | Pearson Correlation | 0.390 | 0.625^*^ | 0.220 | 0.420 | 0.416 | 0.211 | -0.535 | 0.418 | 0.442 | 0.267 | 0.222 | -- |  |  |  |
|  | Sig. (2-tailed) | 0.151 | 0.013 | 0.430 | 0.119 | 0.123 | 0.451 | 0.641 | 0.121 | 0.099 | 0.336 | 0.427 |  |  |  |  |
|  | R^2^ | 0.152 | 0.391 | 0.048 | 0.176 | 0.173 | 0.044 | 0.286 | 0.175 | 0.195 | 0.071 | 0.049 |  |  |  |  |
|  | N | 15 | 15 | 15 | 15 | 15 | 15 | 3 | 15 | 15 | 15 | 15 | 15 |  |  |  |
| *S. marcescens* bacterial resistance | Pearson Correlation | 0.153 | 0.393 | 0.514^*^ | 0.442 | 0.609^*^ | 0.078 | -0.685 | 0.454 | -0.092 | -0.164 | -0.115 | 0.360 | -- |  |  |
|  | Sig. (2-tailed) | 0.586 | 0.147 | 0.050 | 0.099 | 0.016 | 0.782 | 0.520 | 0.089 | 0.745 | 0.558 | 0.682 | 0.187 |  |  |  |
|  | R^2^ | 0.023 | 0.154 | 0.264 | 0.195 | 0.371 | 0.006 | 0.469 | 0.206 | 0.008 | 0.027 | 0.013 | 0.130 |  |  |  |
|  | N | 15 | 15 | 15 | 15 | 15 | 15 | 3 | 15 | 15 | 15 | 15 | 15 | 15 |  |  |
| *S. aureus* bacterial resistance | Pearson Correlation | 0.858^**^ | 0.685^**^ | 0.573^*^ | -0.024 | 0.510 | -0.068 | -0.583 | 0.721^**^ | -0.367 | -0.565^*^ | -0.439 | 0.195 | 0.001 | -- |  |
|  | Sig. (2-tailed) | 0.000 | 0.005 | 0.025 | 0.933 | 0.052 | 0.811 | 0.604 | 0.002 | 0.179 | 0.028 | 0.102 | 0.487 | 0.996 |  |  |
|  | R^2^ | 0.736 | 0.469 | 0.328 | 0.001 | 0.260 | 0.005 | 0.400 | 0.520 | 0.135 | 0.319 | 0.193 | 0.038 | 0.000 |  |  |
|  | N | 15 | 15 | 15 | 15 | 15 | 15 | 3 | 15 | 15 | 15 | 15 | 15 | 15 | 15 |  |
| *S. epidermidis* bacterial resistance | Pearson Correlation | 0.817^**^ | 0.712^**^ | 0.272 | -0.275 | 0.487 | 0.124 | -0.577 | 0.443 | 0.091 | -0.255 | -0.114 | 0.243 | -0.102 | 0.814^**^ | -- |
|  | Sig. (2-tailed) | 0.000 | 0.003 | 0.327 | 0.322 | 0.065 | 0.659 | 0.609 | 0.098 | 0.746 | 0.360 | 0.685 | 0.382 | 0.718 | 0.000 |  |
|  | R^2^ | 0.667 | 0.507 | 0.074 | 0.076 | 0.237 | 0.015 | 0.333 | 0.196 | 0.008 | 0.065 | 0.013 | 0.059 | 0.010 | 0.663 |  |
|  | N | 15 | 15 | 15 | 15 | 15 | 15 | 3 | 15 | 15 | 15 | 15 | 15 | 15 | 15 | 15 |

***Table S10:*** *Correlation matrix, generated by SPSS, of the bacterial resistance and DDD-prescriptions for clarithromycin from 2008-2022. Dark green colour and “**” indicates a significant correlation at the 0.01 level. Light green colour and “*” indicates a significant correlation at the 0.05 level. Orange colour indicates no significant correlation.*

|  | | 10 Clarithromycin DDD-prescriptions | *S. aureus*  bacterial resistance | *S. epidermidis* bacterial resistance | *S. pneumoniae* bacterial resistance |
| --- | --- | --- | --- | --- | --- |
| 10 Clarithromycin  DDD-prescriptions | Pearson Correlation | -- |  |  |  |
|  | N | 32 |  |  |  |
| *S. aureus*  bacterial resistance | Pearson Correlation | 0.752^**^ | -- |  |  |
|  | Sig. (2-tailed) | 0.001 |  |  |  |
|  | R^2^ | 0.565 |  |  |  |
|  | N | 15 | 15 |  |  |
| *S. epidermidis*  bacterial resistance | Pearson Correlation | 0.733^**^ | 0.419 | -- |  |
|  | Sig. (2-tailed) | 0.002 | 0.120 |  |  |
|  | R^2^ | 0.537 | 0.176 |  |  |
|  | N | 15 | 15 | 15 |  |
| *S. pneumoniae*  bacterial resistance | Pearson Correlation | 0.358 | 0.012 | 0.197 | -- |
|  | Sig. (2-tailed) | 0.190 | 0.966 | 0.482 |  |
|  | R^2^ | 0.128 | 0.000 | 0.039 |  |
|  | N | 15 | 15 | 15 | 15 |

Supplemental Figures

***Fig. S1****: Correlation panels for the correlations between bacterial resistance and DDD-prescriptions for azithromycin. For the years 2008 to 2022, the development of bacterial resistance is plotted on the x-axis and DDD-prescriptions are plotted on the y-axis.*
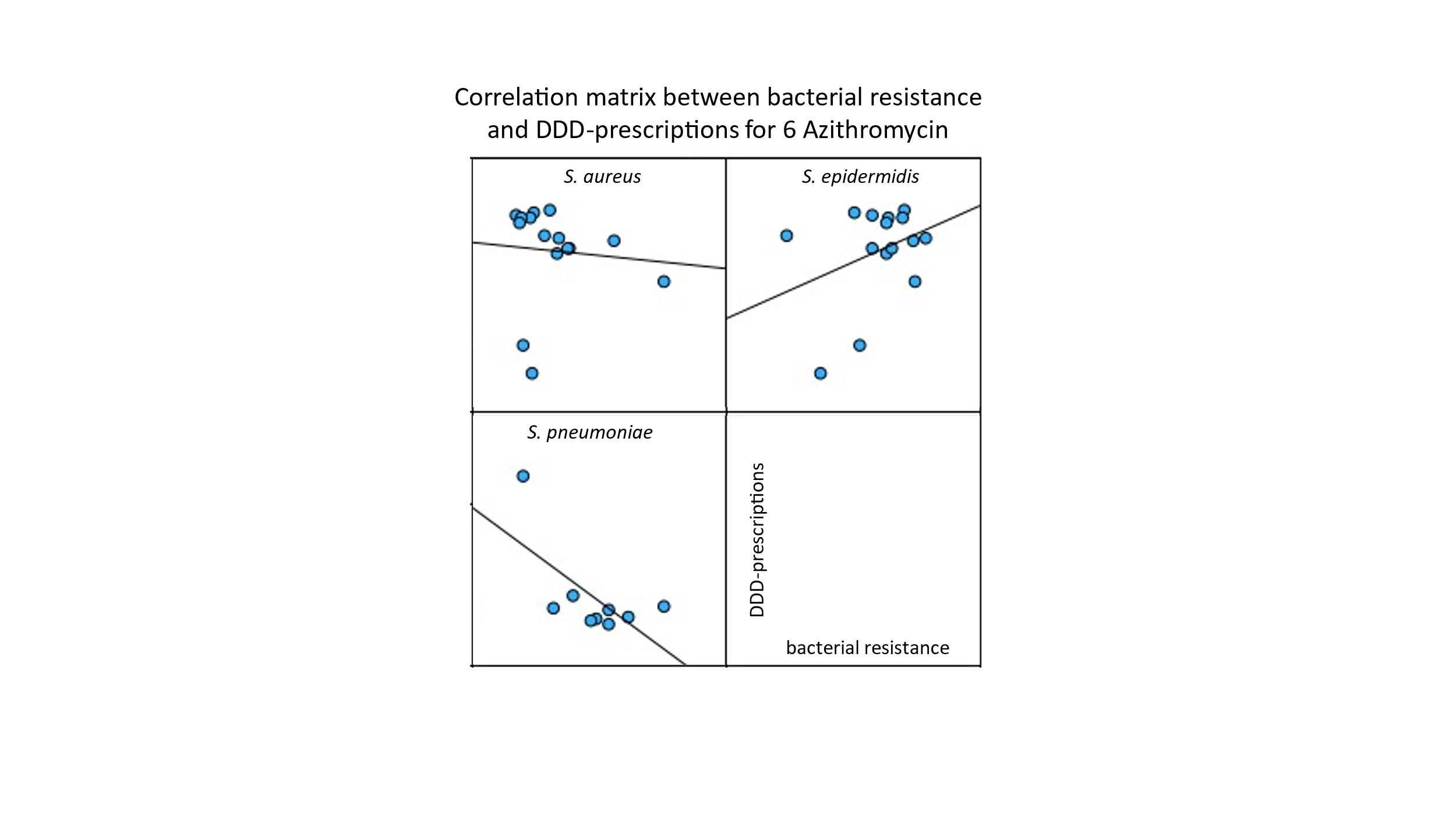


***Fig. S2****: Correlation panels for the correlations between bacterial resistance and DDD-prescriptions for sulfamethoxazole-trimethoprim. For the years 2008 to 2022, the development of bacterial resistance is plotted on the x-axis and DDD-prescriptions are plotted on the y-axis.*

*
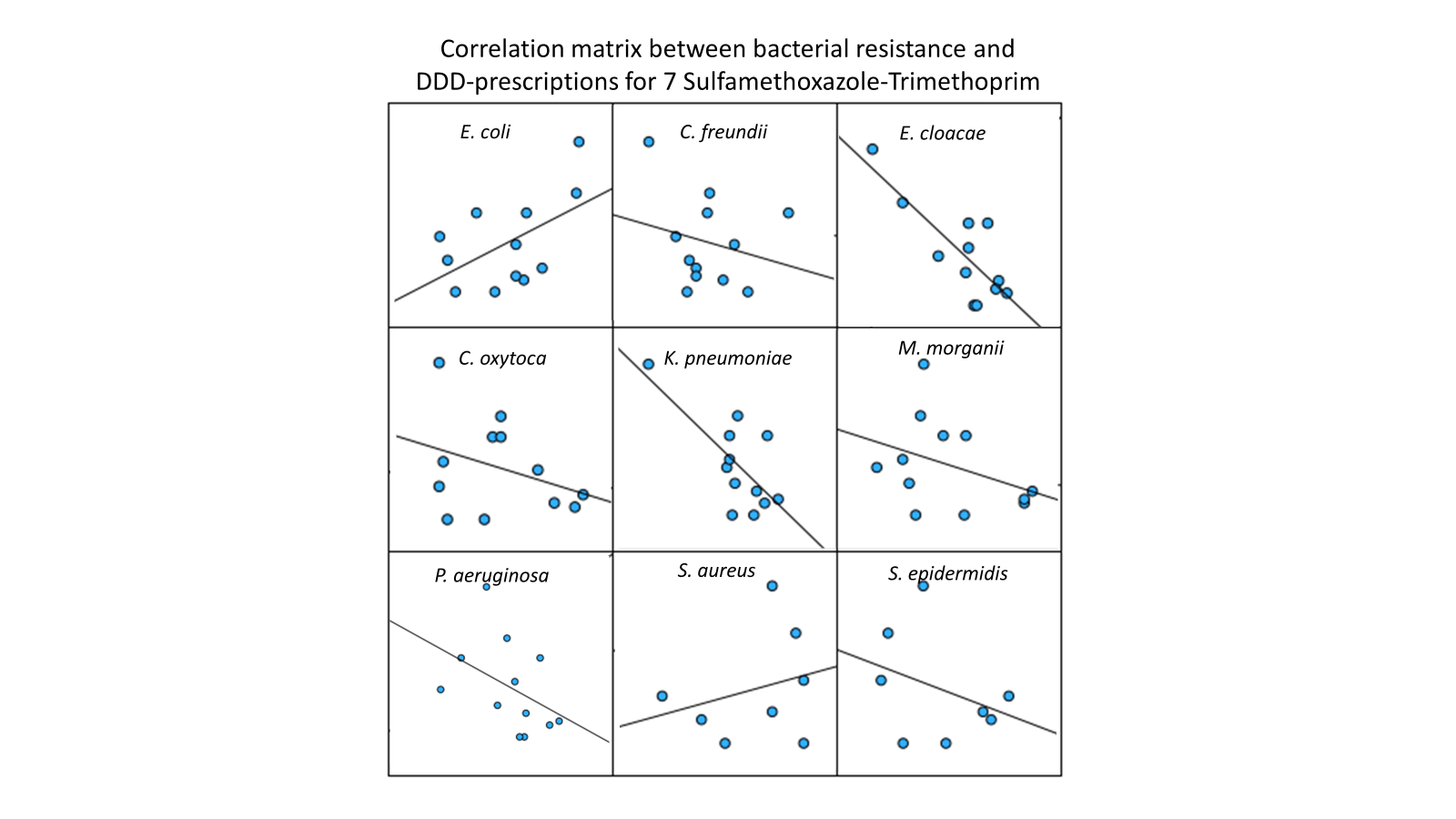
*

***Fig. S3****: Correlation panels for the correlations between bacterial resistance and DDD-prescriptions for nitrofurantoin. For the years 2008 to 2022, the development of bacterial resistance is plotted on the x-axis and DDD-prescriptions are plotted on the y-axis.*

*
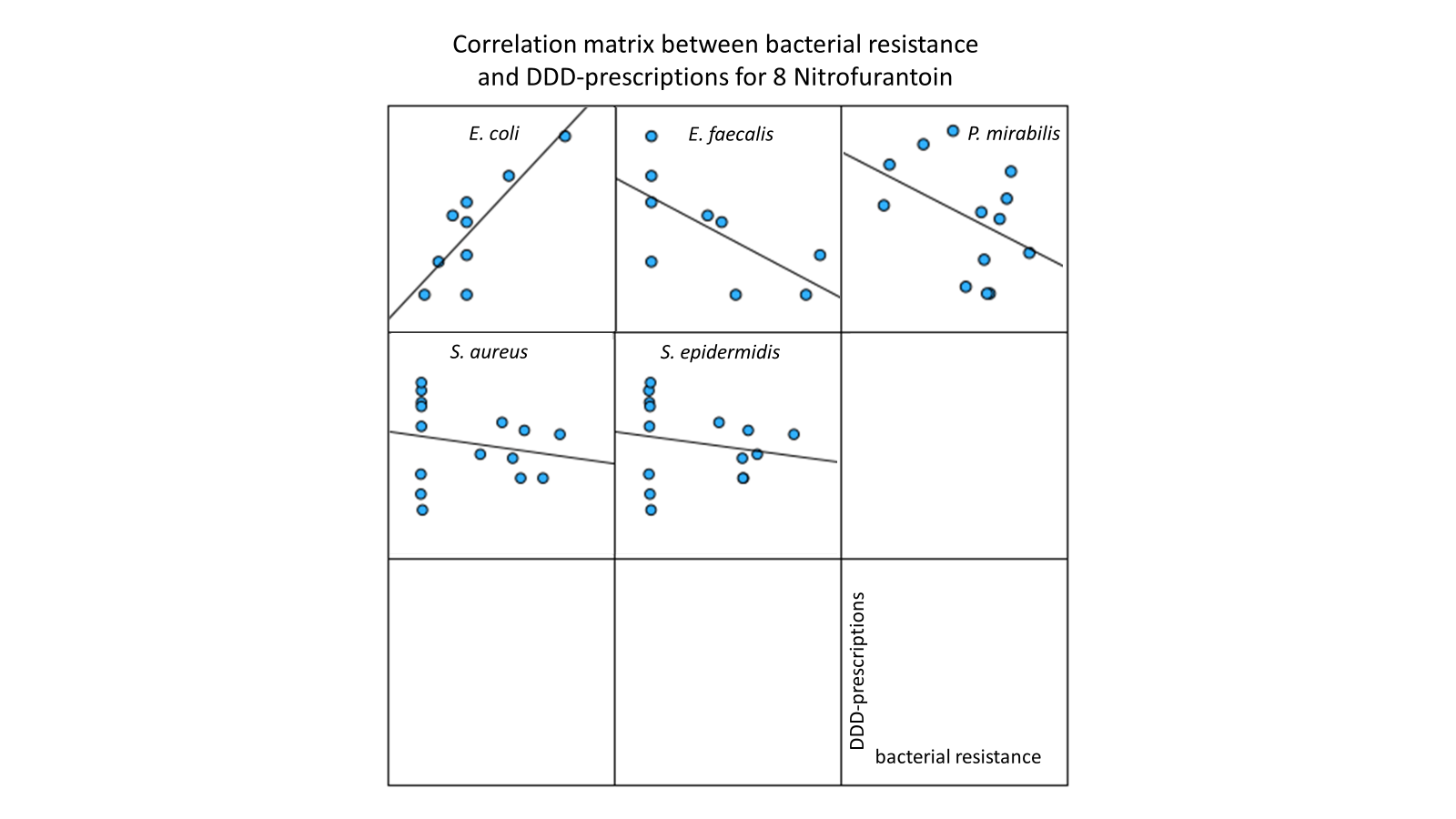
*

***Fig. S4****: Correlation panels for the correlations between bacterial resistance and DDD-prescriptions for ciprofloxacin. For the years 2008 to 2022, the development of bacterial resistance is plotted on the x-axis and DDD-prescriptions are plotted on the y-axis.*

*
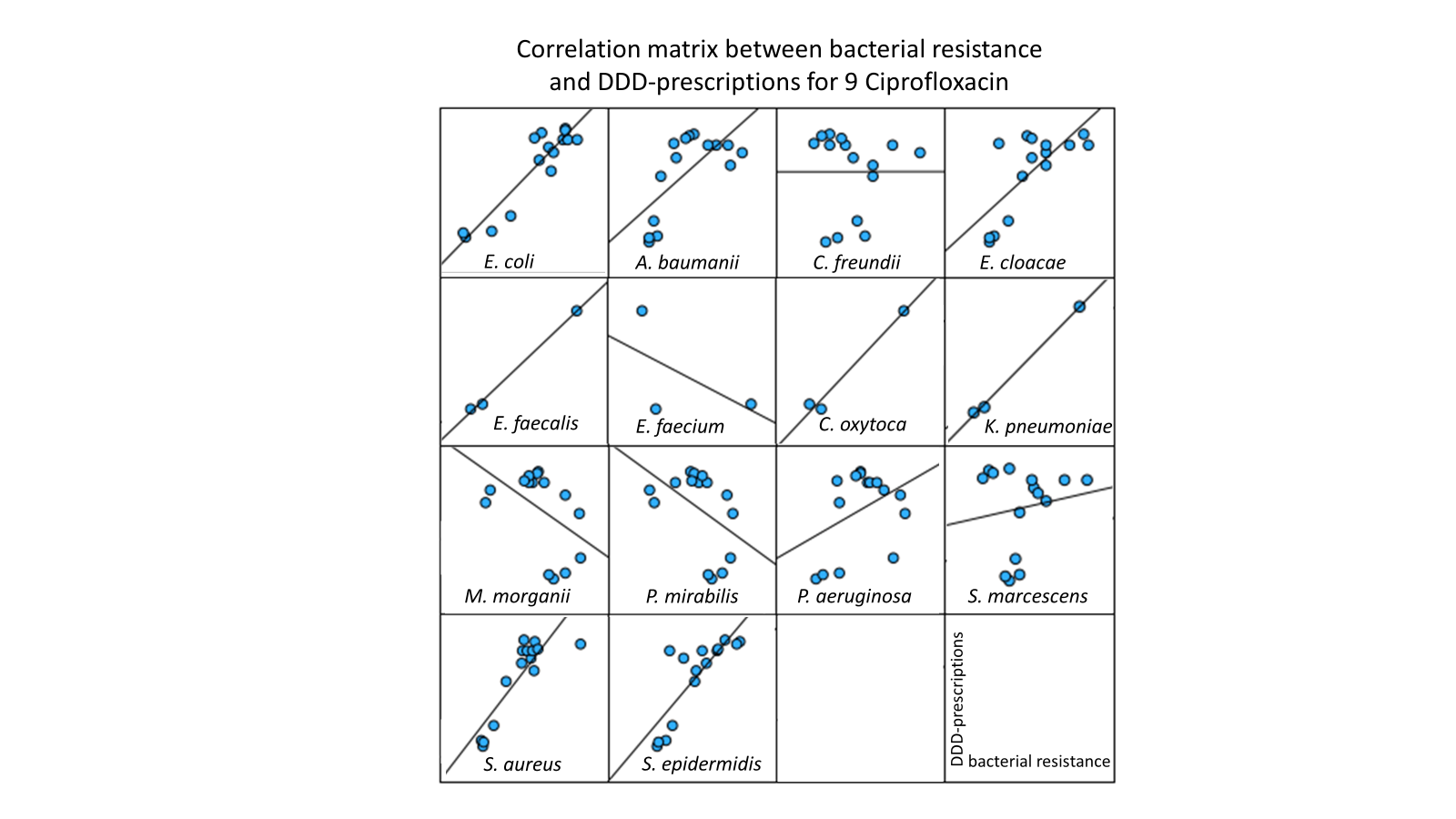
*

***Fig. S5****: Correlation panels for the correlations between bacterial resistance and DDD-prescriptions for clarithromycin. For the years 2008 to 2022, the development of bacterial resistance is plotted on the x-axis and DDD-prescriptions are plotted on the y-axis.*

*
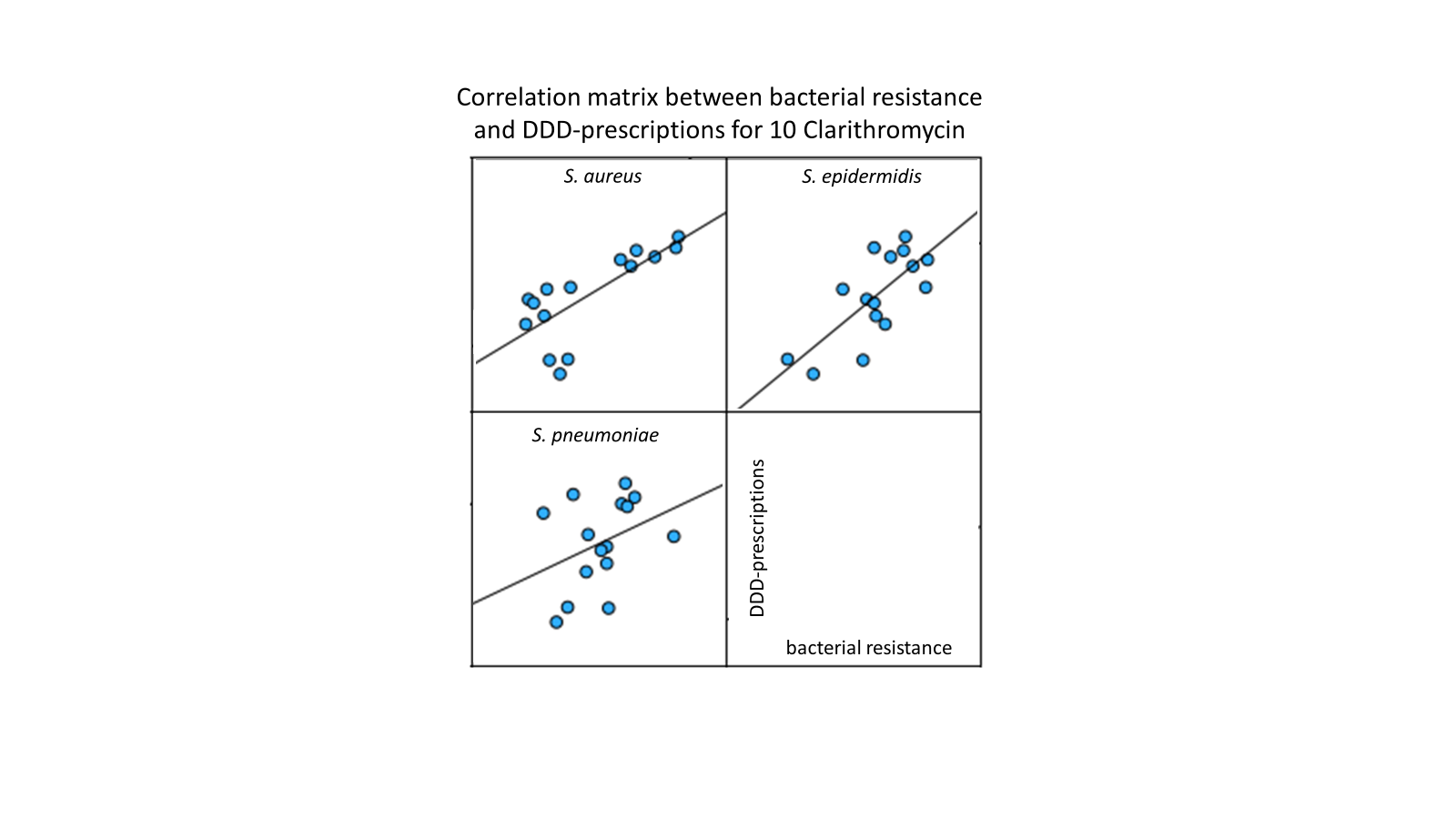
*
